# Approximate conditional phenotype analysis based on genome wide association summary statistics

**DOI:** 10.1038/s41598-021-82000-1

**Published:** 2021-01-28

**Authors:** Peitao Wu, Biqi Wang, Steven A. Lubitz, Emelia J. Benjamin, James B. Meigs, Josée Dupuis

**Affiliations:** 1 Department of Biostatistics, Boston University School of Public Health, Boston, MA USA; 2 Cardiac Arrhythmia Service, Massachusetts General Hospital, Boston, MA USA; 3 The Broad Institute of Harvard and MIT, Cambridge, MA USA; 4 Cardiology and Preventive Medicine Sections, Evans Department of Medicine, Boston University School of Medicine, Boston, MA USA; 5 National Heart, Lung, and Blood Institute’s and Boston University’s Framingham Heart Study, Framingham, MA USA; 6 Department of Epidemiology, Boston University School of Public Health, Boston, MA USA; 7 Division of General Internal Medicine, Massachusetts General Hospital, Boston, MA USA; 8 Harvard Medical School, Boston, MA USA

**Keywords:** Genome-wide association studies, Statistical methods

## Abstract

Because single genetic variants may have pleiotropic effects, one trait can be a confounder in a genome-wide association study (GWAS) that aims to identify loci associated with another trait. A typical approach to address this issue is to perform an additional analysis adjusting for the confounder. However, obtaining conditional results can be time-consuming. We propose an approximate conditional phenotype analysis based on GWAS summary statistics, the covariance between outcome and confounder, and the variant minor allele frequency (MAF). GWAS summary statistics and MAF are taken from GWAS meta-analysis results while the traits covariance may be estimated by two strategies: (i) estimates from a subset of the phenotypic data; or (ii) estimates from published studies. We compare our two strategies with estimates using individual level data from the full GWAS sample (gold standard). A simulation study for both binary and continuous traits demonstrates that our approximate approach is accurate. We apply our method to the Framingham Heart Study (FHS) GWAS and to large-scale cardiometabolic GWAS results. We observed a high consistency of genetic effect size estimates between our method and individual level data analysis. Our approach leads to an efficient way to perform approximate conditional analysis using large-scale GWAS summary statistics.

## Introduction

Genome-wide association studies (GWAS) have been successful in identifying the associations between genetic variants and complex traits. Because genetic variants may have pleiotropic effects, one trait can be a confounder in a GWAS to identify loci associated with another trait. A typical approach to address the confounding issue is to test the association between the trait and a genetic variant adjusting for the confounders. Association results may vary due to confounding, so further adjustment for potential confounders in GWAS is crucial. Moreover, adjusting for traits that explain a large proportion of the variance may increase power to detect genetic associations by reducing the variance of the adjusted traits. For example, Dupuis et al. 2010 conducted a GWAS of fasting insulin (FI) without adjustment for body mass index (BMI) and identified two loci (*GCKR, IGF1*) associated with FI^1^. Manning et al. 2012 also conducted a GWAS meta-analysis of FI and additionally identified 6 previously unreported loci (*COBLL1-GRB14, IRS1, PPP1R3B, PDGFC, UHRF1BP1,* and *LYPLAL1*) after adjusting for BMI^2^. Conducting a sensitivity analysis for GWAS by additionally adjusting for one or more traits may lead to new findings. However, in analyses in which many studies contribute to the final results, as is often the case in consortia-based meta-analyses, asking each study to rerun a genome-wide is time-consuming and potentially prohibitive. Moreover, using GWAS summary statistics has the advantage of protecting personal identifiable information and making data sharing possible without violating the participants’ privacy.

We propose a method to evaluate genetic associations adjusting for a confounder using summary statistics from GWAS meta-analysis and covariance estimates between the trait of interest and the confounding trait. We allow the trait of interest and the potential confounder to be either continuous or binary.

An approximate conditional analysis approach has been proposed earlier by Yang et al. 2012 to evaluate the association between a trait and a single nucleotide polymorphism (SNP) adjusting for other SNPs using summary statistics from GWAS and linkage disequilibrium (LD) estimates between SNPs^3^. To extend the conditional analysis adjusting for SNPs to conditional analysis adjusting for another traits (i.e., confounders), in 2017 Deng and Pan proposed an approach to perform approximate conditional analysis to adjust for continuous confounders^4^. However, their method can only be applied to quantitative traits. Zhu et al. 2018 proposed a method to estimate the genetic effects of genetic variants on disease adjusting for other risk factors by integrating Mendelian randomization of summary GWAS statistics and LD-score regressions to approximate the covariance between the trait of interest and the risk factors^5^. This method is applicable to both continuous and binary traits. In addition, Wolf et al. 2020 proposed an approach for continuous outcomes using summary statistics of outcomes and covariates derived from the same study^6^.

Our proposed approach differs from the above-mentioned methods that use genotype data to estimate the covariance between phenotypic traits. We propose estimating the covariance directly from the phenotype data. For example, in order to estimate the covariance between FI and BMI, the best approach would be to use all the available phenotypic data for FI and BMI. However, gathering the full phenotype data in a large consortium is challenging, and confidentiality restrictions often prohibit sharing individual level data. Alternative approaches to estimate the covariance include: (1) evaluating covariance in a subset of the full samples (e.g. estimating the covariance between traits from one cohort in a multi-cohort study); and (2) using a covariance estimate from published articles.

We evaluate our approximate conditional analysis approach and compare the results to the gold standard (conditional analysis using individual level data) using a simulation study. To illustrate results of the approach, we apply our method to cardiometabolic traits studied in one cohort, the Framingham Heart Study (FHS), and in meta-analysis results from several large-scale cardiometabolic GWAS consortia. We selected traits and outcomes that are substantially influenced by one or more secondary traits. In FHS we evaluated anthropometric traits including waist circumference adjusted for BMI, or BMI adjusted for ever-smoking, and cardiac traits including atrial fibrillation adjusted for height or adjusted for both heart failure and myocardial infarction. In large-scale cardiometabolic GWAS consortia meta-analyses, we compared our method with existing approaches using results from multiple traits, including fasting insulin adjusted for BMI, BMI adjusted for ever-smoking, and atrial fibrillation adjusted for BMI or adjusted for coronary artery disease.

**Figure 1:**
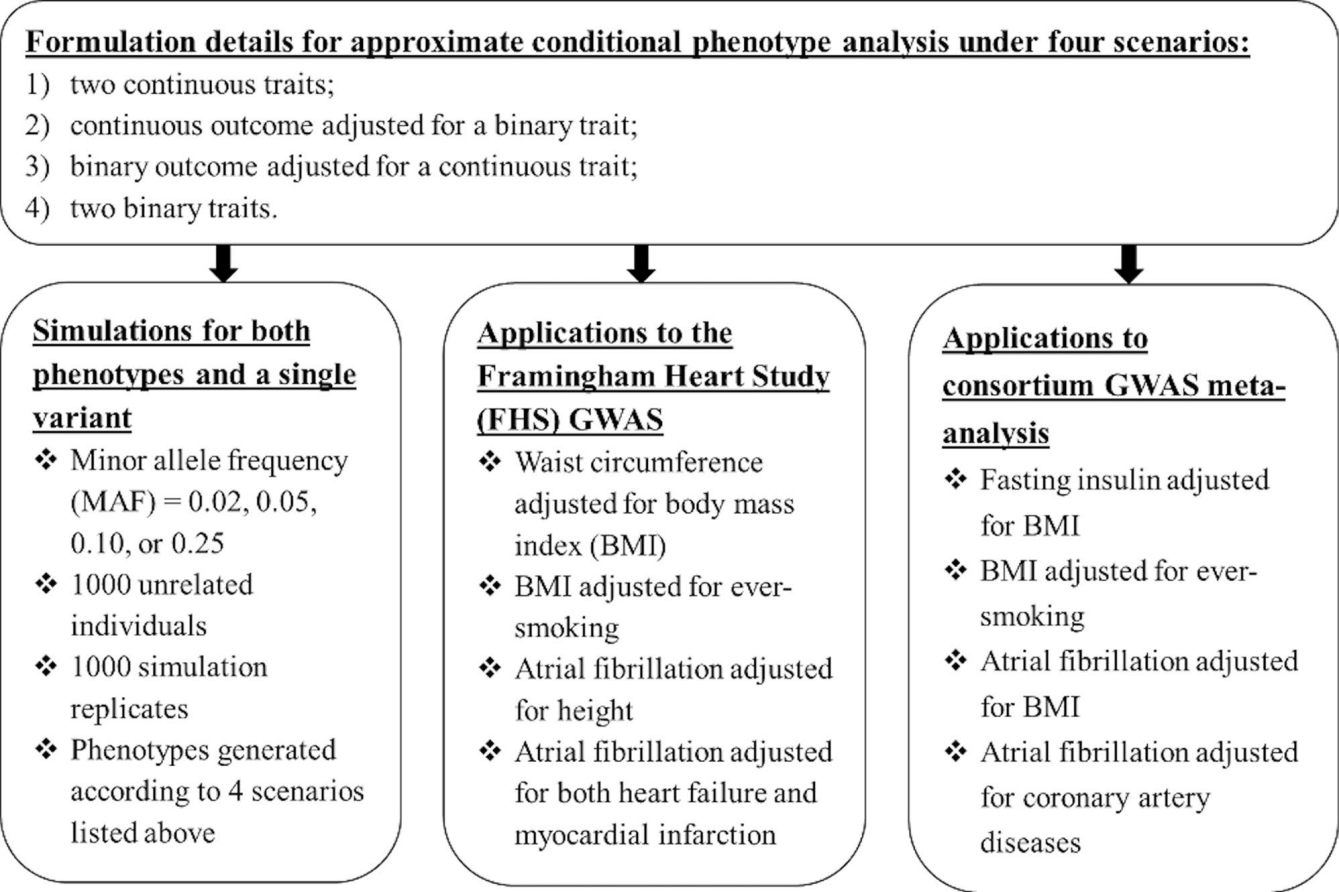
Framework for approximate conditional phenotype analysis evaluation.

The rest of this article is organized as follows. We present simulation results comparing our approach to the gold standard. We then follow with applications to real data sets from FHS and consortium GWAS meta-analyses. In the Method section, we present the formulation details of our new approximate conditional phenotype analysis for the following four scenarios: (1) two continuous traits; (2) continuous outcome adjusted for a binary trait; (3) binary outcome adjusted for a continuous trait; and (4) two binary traits. Our investigation framework is presented in Fig. 1.

## Results

### Simulation results

We compare our proposed method to the gold standard (using individual level data to estimate the genetic variant effect, $\beta$, and its statistical significance). As shown in Table 1, our proposed method performs well in estimating both the effect size (beta) and its standard error for MAF = 2%, 5%, 10%, and 25% when ${\mathbf{Y}}_{1}$ and ${\mathbf{Y}}_{2}$ are continuous, ${\mathbf{Y}}_{1}$ is continuous and ${\mathbf{Y}}_{2}$ is binary, and ${\mathbf{Y}}_{2}$ is continuous and ${\mathbf{Y}}_{1}$ is binary. Our method also yields good performance when the two traits are binary with MAF = 25%. However, the estimates of $\beta$ are less accurate compared to the gold standard when MAF = 2% or 5% for two binary traits ($|{\text{mean}}(\hat{\beta }_{{\text{gold standard}}} ) - {\text{mean}}(\hat{\beta }_{{\text{our method}}} )|/{\text{mean}}(\hat{\beta }_{{\text{gold standard}}} ) \approx 10\%$). Supplementary Figs. 1 to 8 present scatter plots for beta estimates and p-values comparing our method with the gold standard. In addition, in our simulations, when varying the proportion of variance explained by the adjustment covariate from 20 to 2%, we find that the variance explained did not have much impact on the accuracy of the approximation as shown in Table 1 and Supplemental Table 1. We also find a slight upward bias in effect size and standard error estimations when the correlation is up to 20% lower than the true value (i.e., uniformly generate from 80 to 100% of true correlations), while a downward bias is observed when the correlation is up to 20% above the true value for continuous outcomes (i.e., uniformly generate from 100 to 120% of true correlations). For binary outcomes, there is a downward bias in effect size and its standard error estimations whenever the correlation is under or overestimated up to 20% (Supplementary Table 2).

**Table 1:** Simulation results for genetic effect estimation of our method and the gold standard.

MAF	Individual level data frequency (gold standard)		Proposed method full dataset		Proposed method subset dataset (20%)		Proposed method within ± 20% of true value	
	$\hat{\beta }$	SE($\hat{\beta }$)	$\hat{\beta }$	SE($\hat{\beta }$)	$\hat{\beta }$	SE($\hat{\beta }$)	$\hat{\beta }$	SE($\hat{\beta }$)
**Continuous **${\mathbf{Y}}_{1}$** and continuous **${\mathbf{Y}}_{2}$								
2%	0.710	0.146	0.710	0.146	0.709	0.145	0.709	0.145
5%	0.460	0.092	0.460	0.092	0.461	0.092	0.460	0.092
10%	0.332	0.065	0.332	0.065	0.331	0.065	0.332	0.065
25%	0.231	0.046	0.231	0.046	0.23	0.046	0.231	0.046
**Continuous **${\mathbf{Y}}_{1}$** and binary **${\mathbf{Y}}_{2}$								
2%	0.709	0.143	0.707	0.143	0.708	0.143	0.707	0.143
5%	0.460	0.091	0.455	0.091	0.457	0.09	0.455	0.090
10%	0.333	0.064	0.329	0.064	0.329	0.064	0.328	0.064
25%	0.228	0.046	0.222	0.046	0.223	0.046	0.221	0.046
**Continuous **${\mathbf{Y}}_{2}$** and binary **${\mathbf{Y}}_{1}$								
2%	0.597	1.072	0.561	1.101	0.571	1.144	0.541	1.057
5%	0.752	0.288	0.722	0.284	0.727	0.309	0.695	0.277
10%	0.809	0.203	0.781	0.200	0.782	0.215	0.750	0.192
25%	0.862	0.150	0.843	0.149	0.843	0.155	0.811	0.143
**Binary **${\mathbf{Y}}_{1}$** and binary **${\mathbf{Y}}_{2}$								
2%	0.867	0.364	0.786	0.340	0.784	0.342	0.784	0.340
5%	0.886	0.234	0.808	0.219	0.806	0.222	0.806	0.220
10%	0.842	0.172	0.782	0.162	0.784	0.165	0.780	0.162
25%	0.764	0.133	0.744	0.130	0.748	0.132	0.742	0.130

Number in the table represent averages over all simulation replicates.

MAF: minor allele frequency. Individual level data analysis is the gold standard for estimation. “Full dataset” means the relationship between the outcome and the covariate is estimated in the full sample of individuals, but the effect is estimated using our approximate approach. “Subset dataset” means the relationship between the traits is estimated by randomly selecting 200 individuals, or 20% of the total sample size. “Proposed method within ± 20% of true value” means the relationship between the outcome, and the covariate is a random estimate falling with 20% the true covariance between the traits. The latter scenario reflects what might happen when using estimates from published reports.

**Table 2:** Details of the estimation of the trait relationship using our method and GCTA_mtCOJO.

	Our method	GCTA_mtCOJO
Parameters	Not required	Significance level for selecting GWAS signals for instrumental variable. Typically set to 5 × 10^–8^ for most analysis. We reduce this threshold to 5 × 10^–6^ for the BMI GWAS adjusted for ever-smoking
External data	FHS phenotype at the time closest to DNA draw	Genotype data from FHS unrelated individuals are used as LD reference panel LD-score regression results from European population based on 1,000 Genomes for the outcome

Results from our evaluation of type I error and power are shown in Supplementary Table 3–8. We do not observe any inflation of the type I error in the scenario when the SNP and confounder are not associated. In the second scenario when there is an association between the SNP and confounder, inflation is only observed when the two traits are continuous and the correlation is estimated using a subset of individuals or using a correlation estimate from a prior study, which was mimicked by using a randomly generated estimate within ± 20% of true value. To further explore the possible causes of the observed inflation, Supplementary Tables 9–10 indicate that when we increased the ratio of subset sample set to full data set to estimate the relationship between the traits or restrict our literature estimate for the correlation between the covariate and the outcome to be more accurate, the inflation is reduced. The results of the power simulation demonstrate that our proposed approach gains similar power as the gold standard by inclusion of a covariate unassociated with the SNP but explaining a substantial proportion of the variance in the outcome.

### Application to the Framingham heart study

**Figure 2:**
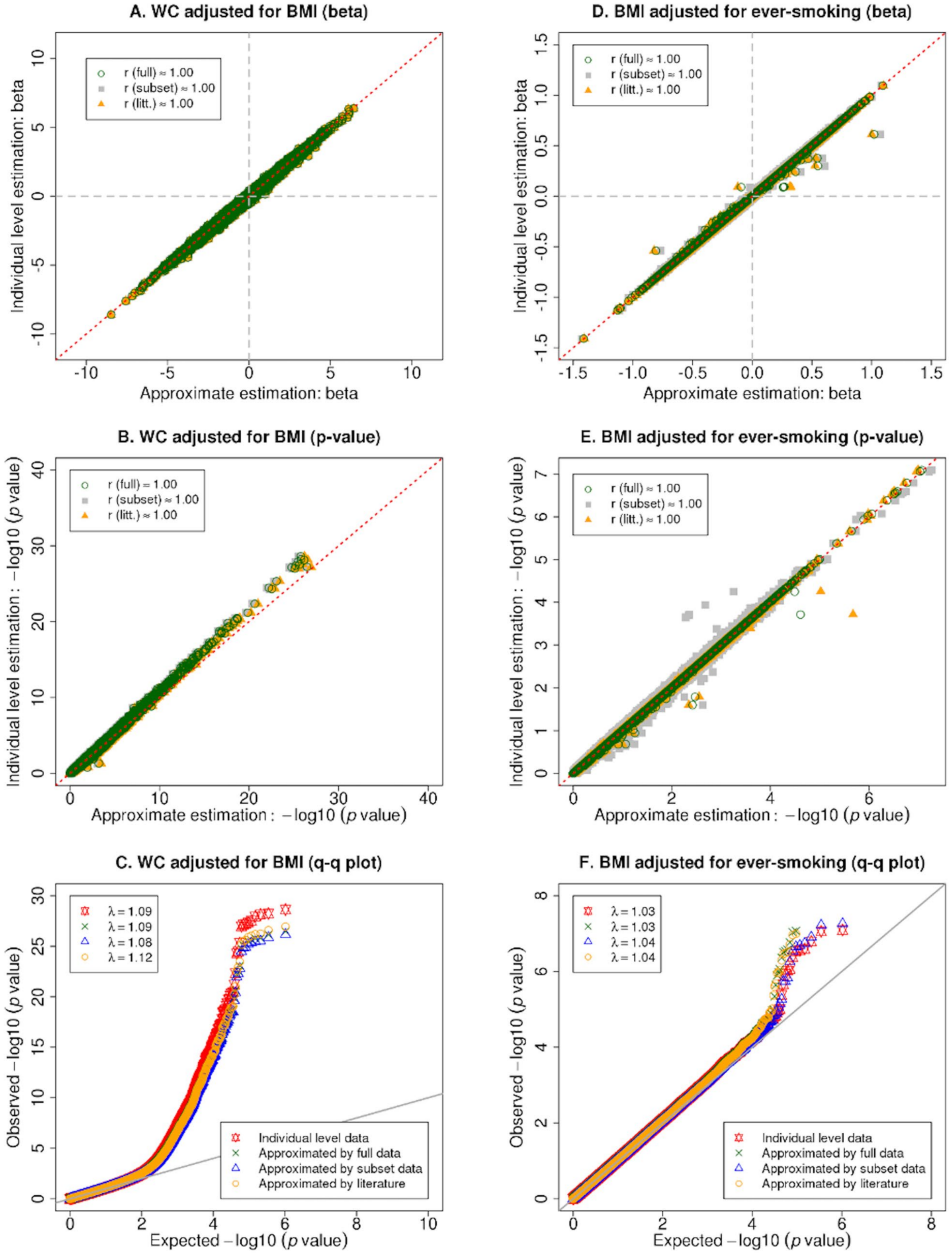
Estimated effect sizes, − log10 (p-values) and quantile–quantile plots for GWAS with continuous outcomes measured in the Framingham Heart Study. Panels (**A**–**C**) present the estimated effect sizes, − log10 (p-values), and quantile–quantile plot (q-q plot), respectively, for GWAS results from analyzing waist circumference (WC) adjusted for body mass index (BMI); panels (**D**–**F**) present the estimated effect sizes, − log10 (p-values), and q-q plot, respectively, for BMI adjusted for ever-smoking.

Estimated effect sizes and − log10 (p-values), and quantile–quantile plots for the FHS GWAS results are displayed in Fig. 2. When the outcome is continuous (WC or BMI), our method yields estimates with high consistency compared to the gold standard (estimates obtained from individual level data); the correlation coefficients (r) between the approximate effect sizes and the gold standard effect sizes are approximately equal to 1 for both continuous (BMI) and binary (ever-smoking) covariates. In addition, for continuous outcomes, the type I error rate is well controlled. For the top WC GWAS hits adjusted for BMI, our method was more conservative compared to the estimates from individual level data (Fig. 2C).

**Figure 3:**
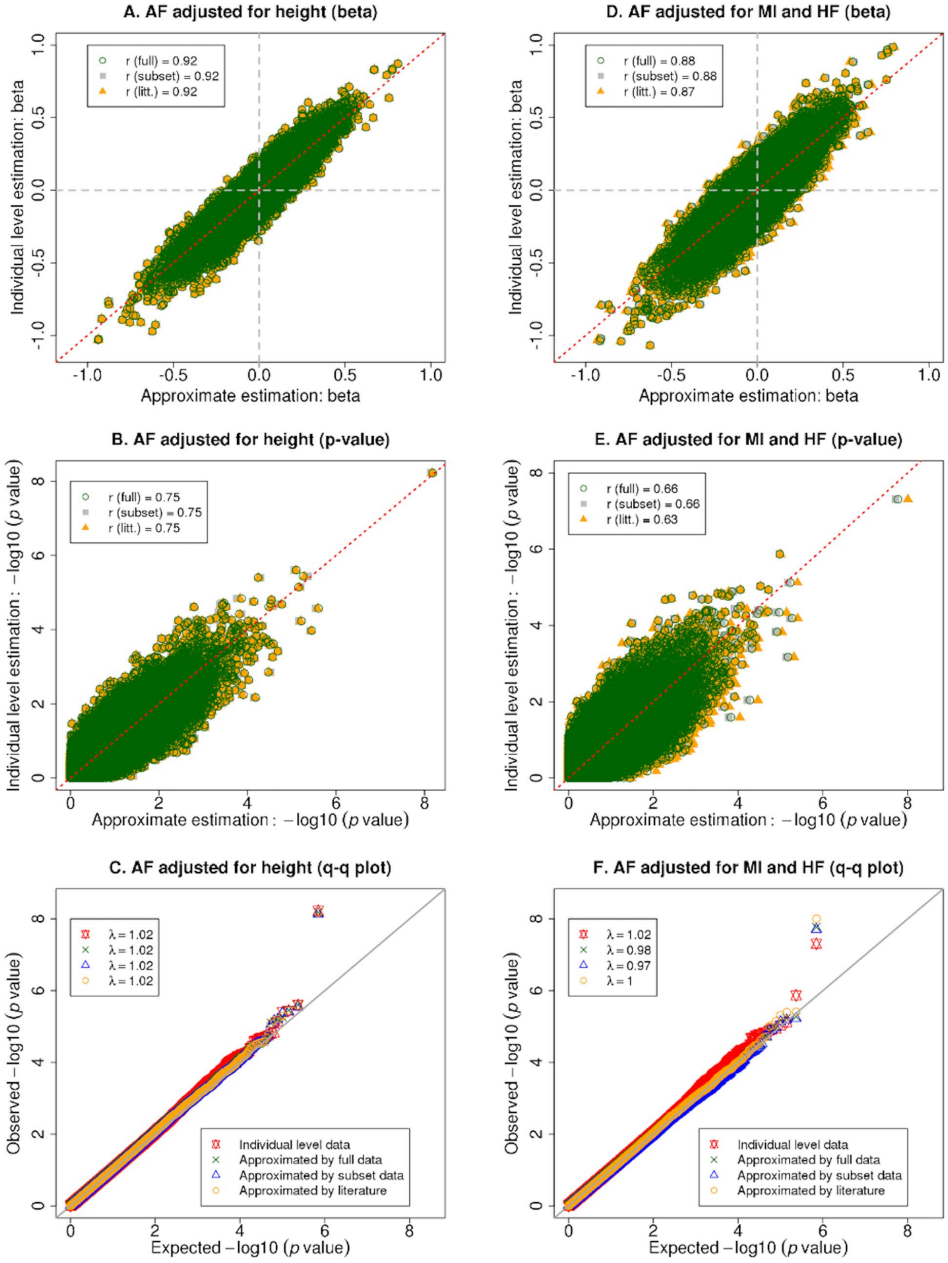
Estimated effect sizes, − log10 (p-values) and quantile–quantile plots for GWAS results with binary outcomes measured in the Framingham Heart Study. Panels (**A**–**C**) present estimated effect sizes, − log10 (p-values), and quantile–quantile plot (q-q plot), respectively, for GWAS results from analyzing atrial fibrillation (AF) adjusted for height; panels (**D**–**F**) present the estimated effect sizes, − log10 (p-values), and q-q plot, respectively, for AF adjusted for both myocardial infarction (MI) and heart failure (HF). Individual level data analysis is the gold standard for estimation, “full” means the relationship between the outcome and the covariate is estimated using a full sample of individuals, “subset” means the relationship is estimated using a random subset of 1,000 individuals, and “litt.” or literature means the relationship is taken from published reports^10,11^.

When the outcome is binary (AF), our approximate approach does not perfectly match estimates from individual level data (the correlation coefficient between the approximation and gold standard for the effect estimates (betas) ranges from 0.87 to 0.92, while correlation coefficient for –log10 (p-values) ranges from 0.64 to 0.75 (Fig. 3A–E). There is no type I error inflation when the adjustment covariate is continuous (Fig. 3C). However, there is a little deflation when the outcome and adjustment covariates are both binary (genomic lambda = 0.98) when using the full phenotype data to estimate the relationship between the two traits. For the top signals in AF GWAS adjusted for both MI and HF, our method yields smaller p-values compared to the gold standard using individual level estimates (Fig. 3F). Individual level data analysis is the gold standard for estimation, “full” means the relationship between the outcome and the covariate is estimated using the full sample of individuals, “subset” means the relationship is estimated using a random sample of 1,000 individuals, and “litt.” or literature means the relationship is taken from published reports^7–9^.

We compared our method with Wolf et al.’s proposed approximation in FHS using WC GWAS adjusted for BMI (Supplemental Fig. 9 and Supplemental Fig. 10). We found high consistency (correlation > 0.996) for both effect estimates and p values of the two methods.

### Application to publicly available cardiometabolic GWAS meta-analysis results

**Figure 4:**
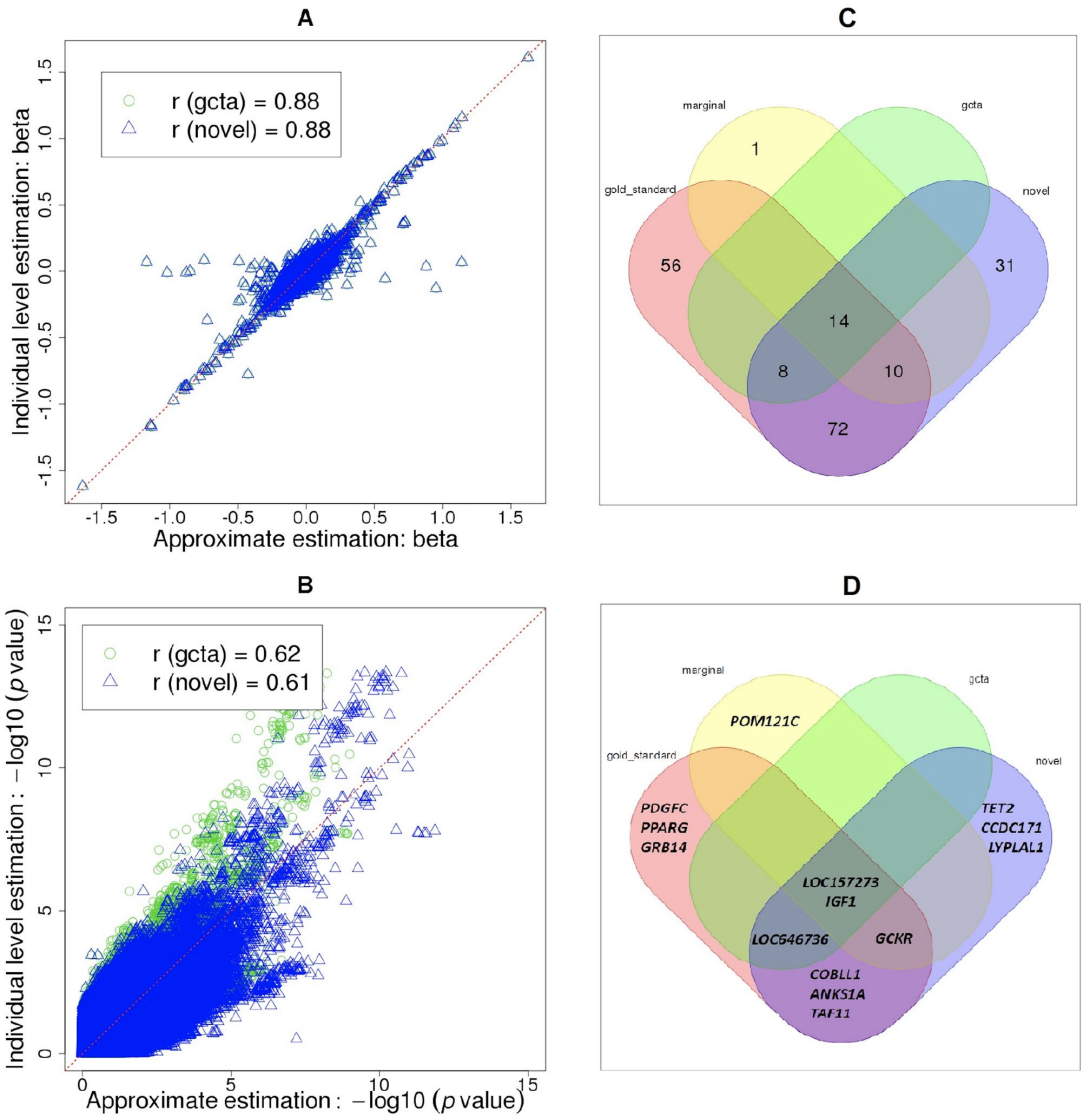
Estimated effect sizes (**A**), − log10 (p-values) (**B**), number of genome-wide significant variants (**C**), and genome-wide significant genes (**D**) for existing GWAS meta-analysis for fasting insulin adjusted for body mass index. Individual level data results provide the gold standard for estimation and is denoted as “gold_standard” in the Venn diagram, “marginal” results are the fasting insulin GWAS results without BMI adjustment, and “gcta” results are obtained using multi-trait-based conditional and joint analysis (mtCOJO) implemented in GCTA 1.9 (GCTA mtCOJO), with the Framingham Heart Study (FHS) unrelated subset of individuals used for the LD reference panel. “Novel” results are obtained from our novel method with phenotype data from FHS to estimate the relationship between traits. Genome-wide significant level equals to the 0.05/total number of variants (0.05/2,407,460 = 2.08 × 10^–8^). Genome-wide significant genes are the genes closest to the significant variants.

When the outcome is natural log-transformed fasting insulin (FI) and the adjustment covariate is BMI, the correlation between the gold standard effect estimates and the estimates obtained from our method, with relationship between traits estimated from a subset of individuals, is r = 0.88, very similar to the correlation coefficient obtained from GCTA_mtCOJO (Fig. 4). The effect estimates obtained with GCTA_mtCOJO and our approach are almost identical (r = 0.99). There are only 9 variants with absolute difference of betas greater than 0.5, and all are from rare variants, with effect allele frequencies ranging from 0.8 to 1.3%.

The correlation coefficients of − log10 (p-values) between the gold standard (conditional analysis with individual level data) and our method using a subset of the data to evaluate the relationship between outcome and adjustment covariate (r = 0.61) is similar to the correlation coefficient obtained for the − log10 (p-values) from gold standard versus GCTA_mtCOJO (r = 0.62).

Our approach identifies many more genome-wide significant variants for FI with BMI adjustment than the FI marginal GWAS analysis or the GCTA_mtCOJO approximate BMI adjustment, of which 72 variants have been validated by the gold standard approach (Fig. 4C). In terms of genes closest to those significant variants, we also find three genes (*COBLL1, ANKS1A,* and *TAF11*) which have not been identified by GCTA_mtCOJO or marginal GWAS but have been validated by gold standard results (Fig. 4D).

For other trait applications (BMI adjusted for ever-smoking, AF adjusted for BMI, or AF adjusted for CAD), our method and GCTA_mtCOJO yields very similar results in effect estimates and p-values. Results from these analyses are presented in Supplemental Fig. 11.

In our investigation, we notice that most GWAS studies require data transformation (e.g., inverse normalized transformation) for continuous traits, especially when the continuous trait is the outcome. In order to see the effect of the data transformation, we apply an inverse-normal transformation to the WC residuals in FHS and use the full phenotype data to estimate the relationship between outcome and covariate. Despite high correlation coefficients for effect estimates (r = 0.95), the approximate effect sizes are two times smaller than the individual level data estimates. We also find biased estimates when we use mtCOJO by GCTA from consortium data (r for effect estimates = 0.69) when the trait of interest has been transformed.

Another issue when applying our method to existing GWAS results relates to allele frequency differences between GWAS datasets. This issue is observed when we analyzed FI adjusted for BMI; one variant has a very different allele frequency in the meta-analysis for BMI (MAF = 11.68%) compared to meta-analysis results for FI (MAF = 0.83%). This variant, rs11672564, also has a great discrepancy between the approximate method and gold standard, which can be explained by the effect allele frequency difference between the two datasets. To address this issue, we use the mean allele frequencies or the minimum allele frequency in the two consortia. However, the results do not improve substantially (see Supplement Fig. 12). Filtering variants with significant difference in allele frequencies (p-values less than 0.05 after Bonferroni correction) resolves this issue (see Supplement Fig. 13).

## Discussion

We propose an approximate method to estimate the effect of a variant on a trait of interest when adjusting for another trait using GWAS summary statistics. Our method is applicable to continuous and binary traits and can be applied to analyze a single SNP without requiring the availability of genome-wide results. We show that the variance of the outcome explained by the adjustment covariate does not have much impact on the accuracy of the approximation. We observed that our approximations for binary outcomes are not as good as continuous outcomes based on our simulation and application results, but our approach is a reasonable approximation method when individual level data analysis is not feasible.

Our proposed method and the mtCOJO by GCTA achieve high consistency in applications to GWAS summary statistics based on consortia. Because our method does not depend on input parameters or require two additional external genetic datasets to estimate the relationship between the traits, our approach is more widely applicable and storage efficient, a great advantage as the number of SNPs included in GWAS increases along with the imputation panel density.

Another advantage of our method that merits discussion is the generalization to multiple confounders adjustment. From the FHS application, we accurately approximate for both effect sizes and p-values when the outcome is binary with two binary confounders. Unlike the conservative approximation by mtCOJO implemented in the software GCTA with fasting insulin adjusted for BMI, our method identifies additional significantly associated variants without the need for individual-level data analysis. Moreover, our approach utilizes summary statistics without requiring individual level data, enabling data sharing without patient confidentiality issues.

Given the advantages mentioned above, we recommend using our approach to adjust for additional covariates when analyzing a large number of variants (e.g., candidate genes, sentinel genes or variants from GWAS) because our method is more efficient in data processing and data storage. When analyzing all GWAS variants, results obtained from our method and existing approaches (e.g., GCTA_mtCOJO) for continuous or binary outcomes are similar. However, we recommend our method over GCTA_mtCOJO in situations where there are very few or no genome-wide significant associations from the GWAS for the covariates because of the difficulty in estimating the genetic correlation between outcome and covariate from GWAS summary statistics.

One potential limitation of all conditional approaches is that sometimes further adjustment for a heritable covariate can lead to bias in estimation of genetic effect, unless the genetic variant is not associated with the covariate or the covariate mediates the genetic effect on the outcome^12^. Because the real causal relationships among genetic variants, the covariate, and the outcome are unknown, we suggest reporting the GWAS results with and without the covariate adjustment. Our approximation method can provide covariate adjusted results without requiring additional individual-level data analysis based on the summary statistics. With the adjusted and unadjusted information at hand, we can potentially estimate the bias of including the covariate and interpret the GWAS results more cautiously^12^. For continuous outcomes, Wang et al^13^ provided corrections to filter potentially spurious associations (i.e., false positive associations) using GWAS summary statistics. We utilized their approach when applying our proposed method and removed more than 100 variants which might be false positives in the WC GWAS adjusted for BMI in the FHS (Supplemental Fig. 14).

There are at least some limitations of our method for applications to existing GWAS results. We used a heuristic justification to approximate $\hat{\beta }$ by the right-hand site of (1) when ${\mathbf{Y}}_{1}$ is binary. However, the simulation studies show that the results of our method are similar to the gold standard except when both traits are binary and the variant has low frequency (MAF ≤ 5%). Another limitation relates to data transformation; when the outcome was rescaled or transformed using an inverse normalized transformation, the approximations for the effect estimates or p-values were less precise. Thus, when applying approximation methods to inverse normal transformed or standardized continuous outcomes, we recommend rerunning the analyses using individual level data whenever possible. One other limitation arises when there are large differences in allele frequencies across different consortia GWAS results. In this instance, we recommend applying our method to GWAS of identical ancestries, and to filter out variants with significant difference in allele frequencies (p-values less than 0.05 after Bonferroni correction) in the two GWAS datasets.

Although our method can adjust for multiple covariates simultaneously, the feasibility of including multiple covariates depends on the number of variants analyzed, the number of covariates, and the available computing resources. In a preliminary implementation with continuous outcomes and covariates, the computing time increases somewhat linearly with addition of covariates. Therefore, our method could feasibly be applied to tens of covariates simultaneously if sufficient computing resources are available.

In conclusion, we propose an approximation to adjust estimates of genetic effects for covariates using GWAS summary statistics. Our approach is applicable to both continuous and binary outcomes, and continuous and binary adjustment covariates, and does not require the availability of genome-wide results. Based on simulations and applications, our approach leads to an efficient way to perform approximate conditional phenotype analysis using widely available summary statistics.

## Method

### Proposed method for approximate conditional analysis

When individual level data for two traits ${\mathbf{Y}}_{i} ,i = 1,2$ and a genetic variant ${\mathbf{X}}$ are available for a sample of size n, we write a generalized linear models (M1 & M2) for each trait as a function of the genetic variant as:

$$g_{i} ({\mathbb{E}}({\mathbf{Y}}_{i} )) = \mu_{i} + {\mathbf{X}}\beta_{i} , \, i = 1,2,$$

in which the function $g_{i} (.) = {\text{logit}}$ if ${\mathbf{Y}}_{i}$ is binary, $g_{i} (.) = {\text{identity}}$ if ${\mathbf{Y}}_{i}$ is continuous.

The relationship between ${\mathbf{Y}}_{1}$ and ${\mathbf{Y}}_{2}$ can also be modelled as a generalized linear model (M3):

$$g_{1} ({\mathbb{E}}({\mathbf{Y}}_{1} )) = \gamma_{0} + {\mathbf{Y}}_{2} \gamma_{m} .$$

Finally, we model the trait ${\mathbf{Y}}_{1}$ as a function of the genetic variant ${\mathbf{X}}$ adjusting for ${\mathbf{Y}}_{2}$ with model 4 (M4):

$$g_{1} ({\mathbb{E}}({\mathbf{Y}}_{1} )) = \beta_{0} + {\mathbf{X}}\beta + {\mathbf{Y}}_{2} \gamma .$$

The parameter $\beta$ in M4 is what we want to estimate based on GWAS summary statistics.

#### Continuous ${\mathbf{Y}}_{1}$ and ${\mathbf{Y}}_{2}$

When ${\mathbf{Y}}_{1}$ and ${\mathbf{Y}}_{2}$ are two continuous traits, then $g_{i} (.)$ is the identity function and M1-M4 are ordinary least square linear models (OLS). Based on the ordinary least squares estimator, we can write

1 $$\widehat{{\mathbf{B}}} = \left( {\begin{array}{*{20}c} {\hat{\beta }} \\ {\hat{\gamma }} \\ \end{array} } \right) = \left( {\begin{array}{*{20}c} {{\mathbf{X}}^{T} {\mathbf{X}}} & {{\mathbf{X}}^{T} {\mathbf{Y}}_{2} } \\ {{\mathbf{Y}}_{2}^{T} {\mathbf{X}}} & {{\mathbf{Y}}_{2}^{T} {\mathbf{Y}}_{2} } \\ \end{array} } \right)^{ - 1} \left( {\begin{array}{*{20}c} {{\mathbf{X}}^{T} {\mathbf{X}}} & {\mathbf{0}} \\ {\mathbf{0}} & {{\mathbf{Y}}_{2}^{T} {\mathbf{Y}}_{2} } \\ \end{array} } \right)\left( {\begin{array}{*{20}c} {\hat{\beta }_{1} } \\ {\hat{\gamma }_{m} } \\ \end{array} } \right).$$

We can obtain ${\mathbf{X}}^{T} {\mathbf{X}}$, ${\mathbf{X}}^{T} {\mathbf{Y}}_{1} ,{\mathbf{X}}^{T} {\mathbf{Y}}_{2} ,{\mathbf{Y}}_{1}^{T} {\mathbf{Y}}_{1} ,{\mathbf{Y}}_{2}^{T} {\mathbf{Y}}_{2}$ by the following equations:

2 $${\mathbf{X}}^{T} {\mathbf{X}} \approx 2n \times {\text{MAF}} \times (1 - {\text{MAF}}),$$

3 $$\hat{\beta }_{1} = ({\mathbf{X}}^{T} {\mathbf{X}})^{ - 1} {\mathbf{X}}^{T} {\mathbf{Y}}_{1} ,$$

4 $$\hat{\beta }_{2} = ({\mathbf{X}}^{T} {\mathbf{X}})^{ - 1} {\mathbf{X}}^{T} {\mathbf{Y}}_{2} ,$$

5 $$\hat{\sigma }_{1}^{2} = \frac{1}{n - 1}{\mathbf{Y}}_{1}^{T} ({\mathbf{I}} - {\mathbf{P}}_{{{\mathcal{C}}({\mathbf{X}})}} ){\mathbf{Y}}_{1} = {\mathbf{X}}^{T} {\mathbf{X}}\widehat{{{\text{var}}}}(\hat{\beta }_{1} ),$$

6 $$\hat{\sigma }_{2}^{2} = \frac{1}{n - 1}{\mathbf{Y}}_{2}^{T} ({\mathbf{I}} - {\mathbf{P}}_{{{\mathcal{C}}({\mathbf{X}})}} ){\mathbf{Y}}_{2} = {\mathbf{X}}^{T} {\mathbf{X}}\widehat{{{\text{var}}}}(\hat{\beta }_{2} ),$$

in which MAF is the minor allele frequency of a genetic variant and Eq. (2) holds under Hardy–Weinberg equilibrium (HWE). The projection matrix ${\mathbf{P}}_{{{\mathcal{C}}({\mathbf{X}})}} = {\mathbf{X}}({\mathbf{X^{\prime}X}})^{ - 1} {\mathbf{X^{\prime}}}$, n is the total sample size. The variance of $\widehat{{\mathbf{B}}}$ is estimated by

7 $$\widehat{{{\text{var}}}}(\widehat{{\mathbf{B}}}) = \frac{1}{n - 2}\left( {\begin{array}{*{20}c} {{\mathbf{X}}^{T} {\mathbf{X}}} & {{\mathbf{X}}^{T} {\mathbf{Y}}_{2} } \\ {{\mathbf{Y}}_{2}^{T} {\mathbf{X}}} & {{\mathbf{Y}}_{2}^{T} {\mathbf{Y}}_{2} } \\ \end{array} } \right)^{ - 1} \left( {{\mathbf{Y}}_{1}^{T} {\mathbf{Y}}_{1} - \left( {\begin{array}{*{20}c} {\hat{\beta }} \\ {\hat{\gamma }} \\ \end{array} } \right)^{T} \left( {\begin{array}{*{20}c} {{\mathbf{X}}^{T} {\mathbf{Y}}_{1} } \\ {{\mathbf{Y}}_{2}^{T} {\mathbf{Y}}_{1} } \\ \end{array} } \right)} \right).$$

Because ${\mathbf{X}}^{T} {\mathbf{X}},{\mathbf{X}}^{T} {\mathbf{Y}}_{1} ,{\mathbf{X}}^{T} {\mathbf{Y}}_{2} ,{\mathbf{Y}}_{1}^{T} {\mathbf{Y}}_{1} ,{\mathbf{Y}}_{2}^{T} {\mathbf{Y}}_{2}$ can be estimated from Eqs. (2)–(6) using summary statistics, we only need to estimate $\hat{\gamma }_{m}$ (the coefficient in the model M3 relating Y_1_ to Y_2_) in order to perform a statistical test of the hypothesis H_0_:$\beta$ = 0. For continuous traits, we propose estimating $\hat{\gamma }_{m}$ from M3 with a subset of individual-level phenotype data. In addition, if the relationship between the two traits has been studied in previous publications, possibly in cohorts with similar characteristics, the prior results can be utilized to estimate $\hat{\gamma }_{m}$ and infer $\beta$ in M4. A third option was proposed by Deng and Pan 2017, who approximated $\hat{\gamma }_{m}$ using $cor({\mathbf{Z}}_{1} ,{\mathbf{Z}}_{2} )$, for which ${\mathbf{Z}}_{i} \in$ ℝ^*m*×1^ is a vector of test statistics (beta/SE(beta)) from the unadjusted models testing the association of genome-wide SNPs other than SNP ${\mathbf{X}}$. This method works well only if both traits are quantitative^4^.

#### Continuous Y_1_ and binary Y_2_

When the adjustment trait is binary, M2 becomes a logistic model. However, other models (M1, M3 and M4) remain OLS models. Note that Eqs. (4) and (6) will not hold when M2 is a logistic regression model, so we cannot obtain ${\mathbf{X}}^{T} {\mathbf{Y}}_{2}$ and ${\mathbf{Y}}_{2}^{T} {\mathbf{Y}}_{2}$ directly from summary statistics.

When Y_2_ is binary, our proposed approximation requires knowledge of the number of cases ($n_{1}$) and controls ($n_{0}$) in addition to the total sample size $n = n_{0} + n_{1}$. Using this information, we can calculate ${\mathbf{Y}}_{2}^{T} {\mathbf{Y}}_{2}$ as

8 $${\mathbf{Y}}_{2}^{T} {\mathbf{Y}}_{2} = n_{1} \left( {1 - \frac{{n_{1} }}{n}} \right).$$

To get an estimate of ${\mathbf{X}}^{T} {\mathbf{Y}}_{2}$, we take advantage of the information provided by the genotype frequencies in cases and controls separately. Genotype frequencies stratified by case status, P_ij_ = ${\mathbb{P}}(X = i|Y_{2} = j)$ for i = 0, 1 or 2 and j = 0 (controls) or 1 (cases), may be available, but if not, they can be estimated from available summary statistics. See the Appendix for details.

Using the stratified genotype frequencies in cases and controls, the quantity ${\mathbf{X}}^{T} {\mathbf{Y}}_{2}$ can be approximated by

9 $$n_{1} ({\mathbb{P}}_{11} + 2{\mathbb{P}}_{21} ) - \frac{{n_{1} \left[ {n_{0} ({\mathbb{P}}_{10} + 2{\mathbb{P}}_{20} ) + n_{1} ({\mathbb{P}}_{11} + 2{\mathbb{P}}_{21} )} \right]}}{n}.$$

Finally, we apply Eqs. (1) and (7) to evaluate the approximate effect size of $\hat{\beta }$ and its corresponding variance.

#### Binary Y_1_ and continuous Y_2_

When Y_1_ is binary and Y_2_ is continuous, M1, M3, and M4 are logistic models, while M2 remains an OLS model. In order to estimate the genetic effect size after adjusting for ${\mathbf{Y}}_{2}$, we use Eq. (1) to calculate $\hat{\beta }$. Note that the equality in Eq. (1) is an approximation and no longer an equality because model M1 is no longer an OLS model. In addition, $\hat{\beta }_{1}$ and $\hat{\gamma }_{m}$ are the corresponding beta coefficients from two logistic regressions, M1 and M3. The estimation of ${\text{var}}(\widehat{{\mathbf{B}}})$ can be approximated by:

10 $$\widehat{{{\text{var}}}}(\widehat{{\mathbf{B}}}) = {\mathbf{V}}^{ - 1} {\mathbf{D}}\left( {\begin{array}{*{20}c} {\widehat{{{\text{var}}}}(\hat{\beta }_{1} )} & {\widehat{{{\text{cov}}}}(\hat{\beta }_{1} ,\hat{\gamma }_{m} )} \\ {\widehat{{{\text{cov}}}}(\hat{\beta }_{1} ,\hat{\gamma }_{m} )} & {\widehat{{{\text{var}}}}(\hat{\gamma }_{m} )} \\ \end{array} } \right){\mathbf{DV}}^{ - 1} ,$$

where $${\mathbf{V}} = \left( {\begin{array}{*{20}c} {{\mathbf{X}}^{T} {\mathbf{X}}} & {{\mathbf{X}}^{T} {\mathbf{Y}}_{2} } \\ {{\mathbf{Y}}_{2}^{T} {\mathbf{X}}} & {{\mathbf{Y}}_{2}^{T} {\mathbf{Y}}_{2} } \\ \end{array} } \right)$$, and $${\mathbf{D}} = \left( {\begin{array}{*{20}c} {{\mathbf{X}}^{T} {\mathbf{X}}} & {\mathbf{0}} \\ {\mathbf{0}} & {{\mathbf{Y}}_{2}^{T} {\mathbf{Y}}_{2} } \\ \end{array} } \right)$$. In Eq. (10), the covariance between the estimated parameters, $\hat{\beta }_{1}$and $\hat{\gamma }_{m}$ cannot be obtained directly from summary statistics. Because the score test and Wald test in logistic regression are asymptotically equivalent, we replace the $\widehat{{{\text{corr}}}}(\hat{\beta }_{1} ,\hat{\gamma }_{m} )$ by $\widehat{{{\text{corr}}}}(\hat{\beta }_{1}^{*} ,\hat{\gamma }_{m}^{*} )$, in which $\hat{\beta }_{1}^{*}$ and $\hat{\gamma }_{m}^{*}$ are OLS estimators. Then we can approximate $\widehat{{{\text{cov}}}}(\hat{\beta }_{1} ,\hat{\gamma }_{m} )$ by

11 $$\begin{gathered} \widehat{{{\text{cov}}}}(\hat{\beta }_{1} ,\hat{\gamma }_{m} ) = \widehat{{{\text{corr}}}}(\hat{\beta }_{1} ,\hat{\gamma }_{m} )\sqrt {\widehat{{{\text{var}}}}(\hat{\beta }_{1} )} \sqrt {\widehat{{{\text{var}}}}(\hat{\gamma }_{m} )} \approx \frac{{\widehat{{{\text{cov}}}}(\hat{\beta }_{1}^{*} ,\hat{\gamma }_{m}^{*} )}}{{\sqrt {\widehat{{{\text{var}}}}(\hat{\beta }_{1}^{*} )} \sqrt {\widehat{{{\text{var}}}}(\hat{\gamma }_{m}^{*} )} }}\sqrt {\widehat{{{\text{var}}}}(\hat{\beta }_{1} )} \sqrt {\widehat{{{\text{var}}}}(\hat{\gamma }_{m} )} \hfill \\ \approx \frac{{{\text{var}}(Y_{1} )({\mathbf{X}}^{T} {\mathbf{X}})^{ - 1} {\mathbf{X}}^{T} {\mathbf{Y}}_{2} ({\mathbf{Y}}_{2}^{T} {\mathbf{Y}}_{2} )^{ - 1} \sqrt {\widehat{{{\text{var}}}}(\hat{\beta }_{1} )} \sqrt {\widehat{{{\text{var}}}}(\hat{\gamma }_{m} )} }}{{\sqrt {\frac{1}{n - 1}({\mathbf{X}}^{T} {\mathbf{X}})^{ - 1} \left( {{\mathbf{Y}}_{1}^{T} {\mathbf{Y}}_{1} - {\mathbf{Y}}_{1}^{T} {\mathbf{X}}({\mathbf{X}}^{T} {\mathbf{X}})^{ - 1} {\mathbf{X}}^{T} {\mathbf{Y}}_{1} } \right)} \sqrt {\frac{1}{n - 1}({\mathbf{Y}}_{2}^{T} {\mathbf{Y}}_{2} )^{ - 1} \left( {{\mathbf{Y}}_{1}^{T} {\mathbf{Y}}_{1} - {\mathbf{Y}}_{1}^{T} {\mathbf{Y}}_{2} ({\mathbf{Y}}_{2}^{T} {\mathbf{Y}}_{2} )^{ - 1} {\mathbf{Y}}_{2}^{T} {\mathbf{Y}}_{1} } \right)} }}. \hfill \\ \end{gathered}$$

In Eq. (11), the only statistic that cannot be obtained directly from summary statistics is ${\mathbf{Y}}_{1}^{T} {\mathbf{Y}}_{2}$. Two additional quantities would allow the estimation of ${\mathbf{Y}}_{1}^{T} {\mathbf{Y}}_{2}$: the mean of ${\mathbf{Y}}_{2}$ among cases (${\text{Mean}}(Y_{2} |Y_{1} = 1)$ and among controls (${\text{Mean}}(Y_{2} |Y_{1} = 0)$). These two additional summary statistics are usually available from each cohort, and allow for the estimate of ${\mathbf{Y}}_{1}^{T} {\mathbf{Y}}_{2}$ as follows:

12 $${\mathbf{Y}}_{1}^{T} {\mathbf{Y}}_{2} = n \times \widehat{{{\text{Cov}}}}({\mathbf{Y}}_{1} ,{\mathbf{Y}}_{2} ) = n_{1} \left( {1 - \frac{{n_{1} }}{n}} \right)\left[ {{\text{Mean}}(Y_{2} |Y_{1} = 1) - {\text{Mean}}(Y_{2} |Y_{1} = 0)} \right].$$

#### Binary ${\mathbf{Y}}_{1}$ and ${\mathbf{Y}}_{2}$

When both ${\mathbf{Y}}_{1}$ and ${\mathbf{Y}}_{2}$ are binary traits, M1 to M4 are logistic models. Equation (1) can be used to estimate $\beta$ in M4, where ${\mathbf{Y}}_{2}^{T} {\mathbf{Y}}_{2}$ and ${\mathbf{X}}^{T} {\mathbf{Y}}_{2}$ are calculated using our proposed approximation method from Eqs. (8) and (9). We also use Eq. (10) to estimate the variance of our proposed $\hat{\beta }$, for which $\widehat{{{\text{cov}}}}(\hat{\beta }_{1} ,\hat{\gamma }_{m} )$ is calculated from Eq. (11) in Sect. 4.1.3.

Although the description of our proposed method includes only one confounder (**Y**_2_), the method is easily extended to multiple confounders (${\mathbf{Y}}_{2} , \ldots ,{\mathbf{Y}}_{m}$) if we infer the relationships between outcome and confounders from summary statistics and phenotypic data (see Appendix for details). We apply the multivariable models to the Framingham Heart Study (atrial fibrillation as the outcome, with history of myocardial infarctions and history of heart failure as confounders) as an example to illustrate the approach for multiple confounder adjustment.

### Simulation studies

We perform a simulation study to evaluate the accuracy of our proposed method in estimating $\hat{\beta }$ and its variance. For each of 1000 simulation replicates, we generate 1000 independent individuals. We first generate the genotype (${\mathbf{X}}$) using a random binomial variable with a minor allele frequency ($p$) equal to 0.02, 0.05, 0.10, or 0.25.

The traits are simulated as follows. When ${\mathbf{Y}}_{1}$ and ${\mathbf{Y}}_{2}$ are continuous, we generate **Y**_2_ from the equation ${\mathbf{Y}}_{2} = {\mathbf{X}}\beta_{2} + \varepsilon_{2}$, where $\varepsilon_{2}$ is normally distributed, and $\beta_{2}$ is fixed so that the genotype explains 4% of the variance in ${\mathbf{Y}}_{2}$. We generate ${\mathbf{Y}}_{1}$ based on equation M4, assuming 2% variance of ${\mathbf{Y}}_{1}$ can be explained by the genotype ${\mathbf{X}}$ and 20% can be explained by ${\mathbf{Y}}_{2}$.

When ${\mathbf{Y}}_{1}$ is binary and ${\mathbf{Y}}_{2}$ is continuous, we generate ${\mathbf{Y}}_{2}$ using the same parameters used in the two continuous trait scenario. We generate the binary variable ${\mathbf{Y}}_{1}$ using a latent uniform (0, 1) variable, setting **Y**_1_ = 1 when two conditions are met: 1) the latent variable exceeds the genotype specific thresholds of 0.1 (**X** = 0), 0.2 (**X** = 1) and 0.4 (**X** = 2); and 2) **Y**_2_ exceeds the 20^th^ percentile of the **Y**_2_ distribution.

When ${\mathbf{Y}}_{1}$ is continuous and ${\mathbf{Y}}_{2}$ is binary, we generate ${\mathbf{Y}}_{2}$ using the approach used to generate ${\mathbf{Y}}_{1}$ in the scenario above, without the additional condition on the second trait exceeding a certain threshold. Then we generate ${\mathbf{Y}}_{1}$ based on M4, assuming 2% of the variance in ${\mathbf{Y}}_{2}$ can be explained by the genotype ${\mathbf{X}}$ and 20% of the variance can be explained by ${\mathbf{Y}}_{2}$.

If ${\mathbf{Y}}_{1}$ and ${\mathbf{Y}}_{2}$ are both binary variables, first we generate ${\mathbf{Y}}_{2}$ using the same method and parameters as the scenario with continuous ${\mathbf{Y}}_{1}$ and binary ${\mathbf{Y}}_{2}$. Then we calculate ${\mathbf{Y}}_{2}^{*}$ using ${\mathbf{Y}}_{2}^{*} = {\mathbf{X\beta }}_{2}^{*} + {\mathbf{Y}}_{1} {{\varvec{\upgamma}}}^{*}$, assuming ${{\beta}}_{2}^{*} = 0.8$ and ${{\gamma}}_{2}^{*} = 2.0$. Note that ${\mathbf{Y}}_{2}^{*}$ now is a continuous variable. We then transform ${\mathbf{Y}}_{2}^{*}$ to ${\tilde{\mathbf{Y}}}_{2}$ via

$$\tilde{Y}_{2i} = \frac{1}{{1 + \exp ( - Y_{2i}^{*} )}}.$$

We updated $\tilde{Y}_{2i}$ by adding a random error generated independently from a centered normal distribution with standard deviation equal to 0.1. Finally we convert the continuous traits $\tilde{Y}_{2i}$ to binary traits ${\text{Y}}_{2i}$ using the 80% quantile of ${\tilde{\mathbf{Y}}}_{2} (Y_{2i} = I(\tilde{Y}_{2i} \ge Q_{80\% } ({\tilde{\mathbf{Y}}}_{2} )))$ as the threshold.

In our simulation, we estimate $\gamma_{m}$ in M3 using three different approaches: 1) using the individual level from the full dataset to fit model M3; 2) using a subset of the individual level data (200 out of 1,000) to fit model M3; and 3) generating $\hat{\gamma }_{m}$ from a uniform distribution with support interval $(0.8*{\text{mean}}(\hat{\gamma }_{m}^{*} ),1.2*{\text{mean}}(\hat{\gamma }_{m}^{*} ))$ to mimic the approximate estimation from literature where $\hat{\gamma }_{m}^{*}$ is estimated from full data. Then we compare our method to the gold standard (using individual level data to estimate $\beta$ and its statistical significance).

In addition, we compute the type I error and power of our proposed approaches and compare them to the gold standard. For the type I error, we consider the following two scenarios: (1) genetic variant ${\mathbf{X}}$ is not associated with outcome of interest ${\mathbf{Y}}_{1}$ or the covariate ${\mathbf{Y}}_{2}$; (2) genetic variant is not associated with ${\mathbf{Y}}_{1}$ but is associated with ${\mathbf{Y}}_{2}$. In the second scenario, we generate ${\mathbf{Y}}_{2}$ using the same setting in the coefficients estimating simulations described above.

We assess power assuming ${\mathbf{X}}$ is not associated with the covariate ${\mathbf{Y}}_{2}$. We take the variance of ${\mathbf{Y}}_{1}$ explained by ${\mathbf{Y}}_{2}$ as 5%, 10%, 20%, and 40% (as $\frac{{\gamma^{2} var(Y_{2} )}}{{var(Y_{1} )}} = 0.05, \, 0.1, \, 0.2,{\text{ or 0}}{.4}$). When ${\mathbf{Y}}_{1}$ is continuous, we let $\frac{{2\beta^{2} MAF(1 - MAF)}}{{var(Y_{1} )}} = 1\%$; when ${\mathbf{Y}}_{1}$ is binary, we set $\frac{{2\beta^{2} MAF(1 - MAF)}}{{var(Y_{1} )}} = 8\%$ in order to get the compareable value of power when the outcome is continuous.

### Real data applications

#### Framingham heart study

The Framingham Heart Study (FHS) is an observational community-based longitudinal study, launched in 1948 to assess risk factors for cardiovascular diseases^14–16^. Details of the genotype and phenotype data collection for FHS can be found elsewhere^17^. A subset of FHS participants with available genotypes for approximately 550,000 SNPs was selected for analysis. The phenotypes were measured at the time closest to the DNA collection. Our method was applied to the FHS under four scenarios: (1) the outcome is waist circumference (WC) and the adjustment covariate is BMI; (2) the outcome is BMI and the adjustment covariate is ever-smoking; (3) the outcome is atrial fibrillation (AF) and the adjustment covariate is height; and (4) the outcome is AF and the two adjustment covariates are history of myocardial infarction (MI) and history of heart failure (HF). Age, sex, and the first ten principle components, to account for possible population stratification, are included as covariates in the models.

The gold standard for our method is the GWAS analysis conducted on individual level data. We compare the effect sizes and significance of each SNPs with the approximate estimates using our proposed approach based on GWAS summary statistics. When applying our proposed method, the phenotypes relationships are estimated under the following three scenarios: (1) using the full phenotypes data; (2) using a randomly selected sample of 1,000 individuals from the full phenotypes data; and (3) using published study estimates. Such published reports include the study of Bozeman et al^7^ reporting on the relationship between WC and BMI, the reports from Plurphanswat et al^8^ and Dare et al^9^, describing the relationship between BMI and ever smoking, the report from Alonso et al^10^ on the relationship between AF and height, or the article from Schnabel et al^11^ describing the relationship between AF and MI or HF.

#### Publicly available GWAS meta-analysis results

We download GWAS summary statistics of fasting insulin (FI), BMI, ever-smoking, AF, and coronary artery diseases (CAD) from several consortia: Meta-analysis of Glucose and Insulin-related traits (MAGIC) for FI^1,2^, Genetic Investigation of Anthropometric Traits (GIANT) for BMI^18^, Tobacco and Genetics (TAG) for ever-smoking^19^, Atrial Fibrillation Consortium (AFGen)^20^, and Coronary Artery Disease Genome wide Replication and Meta-analysis plus The Coronary Artery Disease (C4D) Genetics consortium (CARDIoGRAMplusC4D) for CAD^21^. Because some summary statistics are based on Genome Build 36, we use the web provided tool to convert the genome coordinates to Genome Build 37 (https://genome.ucsc.edu/cgi-bin/hgLiftOver) to get the same coordinates for the different assemblies.

We then use the summary statistics and the estimates of relationship between the outcome and the covariate based on FHS phenotypes data, a participating cohort in these consortia, to approximately estimate the GWAS effect after adjustment for one additional covariate: (1) FI adjusted for BMI; (2) BMI adjusted for ever-smoking; (3) AF adjusted for BMI; and (4) AF adjusted for CAD. Among those four applications, we only have the gold standard (individual level data) from MAGIC for FI adjusted for BMI. For the other analyses, GWAS results adjusted for the additional trait are not available for comparison purpose. We also compare our method with the multi-trait-based conditional and joint analysis (mtCOJO) implemented in GCTA 1.9 which leverages GWAS summary statistics to estimate the relationships for both continuous and binary traits. When conducting the analysis by GCTA_mtCOJO, we use unrelated individuals from FHS as the LD reference panel. We compare the effect sizes and − log10 (p-values) obtained from our method, the gold standard, and GCTA_mtCOJO.

The difference between our method and GCTA_mtCOJO results from the way the relationship between the outcome and the covariates is estimated: we directly estimate the relationship based on phenotype data (usually one cohort from a consortium or from published reports), while GCTA_mtCOJO uses a causal variants set and heritability of the outcome to estimate the phenotypes relationship. Details regarding estimation of the relationship between the two traits for both methods are in Table 2.

All our analyses (approximation functions, simulations, and applications) were run using R/3.6.0. For details, see http://sites.bu.edu/fhspl/publications/approximate-conditional-analysis/.

## Supplementary information

Supplementary Information.
