# Supplementary material for: Approximate conditional phenotype analysis based on genome wide association summary statistics
Source: Sci Rep. 2021 Jan 28;11:2518. doi: 10.1038/s41598-021-82000-1 (PMC7843738; doi:10.1038/s41598-021-82000-1)
Supplement: Supplementary file 1 — Supplementary Information. [file 41598_2021_82000_MOESM1_ESM.doc]

**Approximate conditional phenotype analysis based on genome wide association summary statistics**

**SUPPLEMENTARY MATERIAL**

Peitao Wu^1, *^, Biqi Wang^1, *^, Steven A Lubitz^2, 3^, Emelia J. Benjamin^4-6^, James B. Meigs^7^, Josée Dupuis^1^

1. Department of Biostatistics, Boston University School of Public Health, Boston, MA, USA
2. Cardiac Arrhythmia Service, Massachusetts General Hospital, Boston, MA, USA
3. The Broad Institute of Harvard and MIT, Cambridge, MA, USA
4. Cardiology and Preventive Medicine Sections, Evans Department of Medicine, Boston University School of Medicine, Boston, MA, USA
5. National Heart, Lung, and Blood Institute’s and Boston University’s Framingham Heart Study, Framingham, MA, USA
6. Department of Epidemiology, Boston University School of Public Health, Boston, MA, USA
7. Division of General Internal Medicine, Massachusetts General Hospital, Boston, MA and Harvard Medical School, Boston, MA

*These authors contributed equally to this work.

Supplementary Methods

Method for obtaining genotype frequencies stratified by case status.

Let $p_{0}$ be the frequency of the coded allele in the control group. We only assume that Hardy-Weinberg equilibrium (HWE) holds in the control group, because the proportion of affected subjects in a population is small, the degree of deviation from HWE is expected to be stronger in cases than in controls. We denote P_ij_= for i = 0, 1 or 2 and j= 0 (controls) or 1 (cases). Under the HWE assumption, the following holds:

Let $p$ be the overall coded allele frequency in the full sample containing both cases and controls. The relationship among$p$,$p_{0}$, and the allele frequency in cases (,,) is

From M2, we assume a multiplicative model for the genetic effect on the odds ratio scale, i.e.$\text{OR}_{X=1vs.X=0}=\text{OR}_{X=2vs.X=1}=\exp(\beta_{2}).$ Then we can write

We combined equations (1) to (4) with and solved for P_i1_.

Method to adjust for multiple confounders

Our method can be generalized to adjust for multiple confounders. Assuming the total sample size is $n$, the outcome is $\mathbf{Y}_{1}$, confounding trait matrix is $\mathbf{C}=(\mathbf{Y}_{2},\cdots,\mathbf{Y}_{m+1})\in$ℝ*^n×m^* , a genetic variant denoted **X**, we have (m+1) marginal generalized linear models ($M_{1}\text{ to }M_{m+1}$) written as:

Denote the relationship between $\mathbf{Y}_{i}$ and $\mathbf{Y}_{j}$ as model ij ($M_{ij}$):

The regression of outcome $\mathbf{Y}_{1}$ on variant **X** adjusted for **C** provides the model we want to estimate ($M_{F}$):

where $g_{i}(.)=\text{logit}$ if $\mathbf{Y}_{i}$ is binary, $g_{i}(.)=identity$ if $\mathbf{Y}_{i}$ is continuous. Parameter $\beta$ in $M_{F}$ is our target parameter to estimate based on GWAS summary statistics.

The estimation of the genetic effect size and variance is calculated from:

$$\begin{matrix} \widehat{\mathbf{B}}=\left(\begin{matrix} \hat{\beta} \\ \widehat{\mathbf{\gamma}} \end{matrix}\right)=\left(\begin{matrix} \mathbf{X}^{T}\mathbf{X} & \mathbf{X}^{T}\mathbf{C} \\ \mathbf{C}^{T}\mathbf{X} & \mathbf{C}^{T}\mathbf{C} \end{matrix}\right)^{-1}\left(\begin{matrix} \mathbf{X}^{T}\mathbf{X} & \mathbf{0} \\ \mathbf{0} & \text{diag}\left(\mathbf{C}^{T}\mathbf{C}\right) \end{matrix}\right)\left(\begin{matrix} \hat{\beta}_{1} \\ \widehat{\mathbf{\gamma}}_{m} \end{matrix}\right) \\ =\left(\begin{matrix} \mathbf{X}^{T}\mathbf{X} & \mathbf{X}^{T}\mathbf{Y}_{2} & \mathbf{X}^{T}\mathbf{Y}_{3} & \cdots& \mathbf{X}^{T}\mathbf{Y}_{m+1} \\ \mathbf{Y}_{2}^{T}\mathbf{X} & \mathbf{Y}_{2}^{T}\mathbf{Y}_{2} & \mathbf{Y}_{2}^{T}\mathbf{Y}_{3} & \cdots& \mathbf{Y}_{2}^{T}\mathbf{Y}_{m+1} \\ \mathbf{Y}_{3}^{T}\mathbf{X} & \mathbf{Y}_{3}^{T}\mathbf{Y}_{2} & \mathbf{Y}_{3}^{T}\mathbf{Y}_{3} & \cdots& \mathbf{Y}_{3}^{T}\mathbf{Y}_{m+1} \\ \vdots& \vdots& \vdots& \ddots& \vdots\\ \mathbf{Y}_{m+1}^{T}\mathbf{X} & \mathbf{Y}_{m+1}^{T}\mathbf{Y}_{2} & \mathbf{Y}_{m+1}^{T}\mathbf{Y}_{3} & \cdots& \mathbf{Y}_{m+1}^{T}\mathbf{Y}_{m+1} \end{matrix}\right)^{-1}\left(\begin{matrix} \mathbf{X}^{T}\mathbf{X} & 0 & 0 & \cdots& 0 \\ 0 & \mathbf{Y}_{2}^{T}\mathbf{Y}_{2} & 0 & \cdots& 0 \\ 0 & 0 & \mathbf{Y}_{3}^{T}\mathbf{Y}_{3} & \cdots& 0 \\ \vdots& \vdots& \vdots& \ddots& \vdots\\ 0 & 0 & 0 & \cdots& \mathbf{Y}_{m+1}^{T}\mathbf{Y}_{m+1} \end{matrix}\right) \\ \times\left(\begin{matrix} \hat{\beta}_{1} \\ \hat{\gamma}_{12} \\ \hat{\gamma}_{13} \\ \vdots\\ \hat{\gamma}_{1(m+1)} \end{matrix}\right), \end{matrix}$$

$$\begin{matrix} \widehat{\text{var}}(\widehat{\mathbf{B}})=\mathbf{V}^{-1}\mathbf{D}\widehat{\text{var}}\left[(\beta_{1},\widehat{\mathbf{\gamma}}_{m}^{T})^{T}\right]\mathbf{DV}^{-1} \\ =\left(\begin{matrix} \mathbf{X}^{T}\mathbf{X} & \mathbf{X}^{T}\mathbf{Y}_{2} & \mathbf{X}^{T}\mathbf{Y}_{3} & \cdots& \mathbf{X}^{T}\mathbf{Y}_{m+1} \\ \mathbf{Y}_{2}^{T}\mathbf{X} & \mathbf{Y}_{2}^{T}\mathbf{Y}_{2} & \mathbf{Y}_{2}^{T}\mathbf{Y}_{3} & \cdots& \mathbf{Y}_{2}^{T}\mathbf{Y}_{m+1} \\ \mathbf{Y}_{3}^{T}\mathbf{X} & \mathbf{Y}_{3}^{T}\mathbf{Y}_{2} & \mathbf{Y}_{3}^{T}\mathbf{Y}_{3} & \cdots& \mathbf{Y}_{3}^{T}\mathbf{Y}_{m+1} \\ \vdots& \vdots& \vdots& \ddots& \vdots\\ \mathbf{Y}_{m+1}^{T}\mathbf{X} & \mathbf{Y}_{m+1}^{T}\mathbf{Y}_{2} & \mathbf{Y}_{m+1}^{T}\mathbf{Y}_{3} & \cdots& \mathbf{Y}_{m+1}^{T}\mathbf{Y}_{m+1} \end{matrix}\right)^{-1}\left(\begin{matrix} \mathbf{X}^{T}\mathbf{X} & 0 & 0 & \cdots& 0 \\ 0 & \mathbf{Y}_{2}^{T}\mathbf{Y}_{2} & 0 & \cdots& 0 \\ 0 & 0 & \mathbf{Y}_{3}^{T}\mathbf{Y}_{3} & \cdots& 0 \\ \vdots& \vdots& \vdots& \ddots& \vdots\\ 0 & 0 & 0 & \cdots& \mathbf{Y}_{m+1}^{T}\mathbf{Y}_{m+1} \end{matrix}\right) \\ \times\left(\begin{matrix} \widehat{\text{var}}(\hat{\beta}_{1}) & \widehat{\text{cov}}(\hat{\beta}_{1},\hat{\gamma}_{12}) & \widehat{\text{cov}}(\hat{\beta}_{1},\hat{\gamma}_{13}) & \cdots& \widehat{\text{cov}}(\hat{\beta}_{1},\hat{\gamma}_{1(m+1)}) \\ \widehat{\text{cov}}(\hat{\beta}_{1},\hat{\gamma}_{12}) & \widehat{\text{var}}(\hat{\gamma}_{12}) & \widehat{\text{cov}}(\hat{\gamma}_{12},\hat{\gamma}_{13}) & \cdots& \widehat{\text{cov}}(\hat{\gamma}_{12},\hat{\gamma}_{1(m+1)}) \\ \widehat{\text{cov}}(\hat{\beta}_{1},\hat{\gamma}_{13}) & \widehat{\text{cov}}(\hat{\gamma}_{12},\hat{\gamma}_{13}) & \widehat{\text{var}}(\hat{\gamma}_{13}) & \cdots& \widehat{\text{cov}}(\hat{\gamma}_{13},\hat{\gamma}_{1(m+1)}) \\ \vdots& \vdots& \vdots& \ddots& \vdots\\ \widehat{\text{cov}}(\hat{\beta}_{1},\hat{\gamma}_{1(m+1)}) & \widehat{\text{cov}}(\hat{\gamma}_{12},\hat{\gamma}_{1(m+1)}) & \widehat{\text{cov}}(\hat{\gamma}_{13},\hat{\gamma}_{1(m+1)}) & \cdots& \widehat{\text{var}}(\hat{\gamma}_{1(m+1)}) \end{matrix}\right) \\ \times\left(\begin{matrix} \mathbf{X}^{T}\mathbf{X} & 0 & 0 & \cdots& 0 \\ 0 & \mathbf{Y}_{2}^{T}\mathbf{Y}_{2} & 0 & \cdots& 0 \\ 0 & 0 & \mathbf{Y}_{3}^{T}\mathbf{Y}_{3} & \cdots& 0 \\ \vdots& \vdots& \vdots& \ddots& \vdots\\ 0 & 0 & 0 & \cdots& \mathbf{Y}_{m+1}^{T}\mathbf{Y}_{m+1} \end{matrix}\right)\left(\begin{matrix} \mathbf{X}^{T}\mathbf{X} & \mathbf{X}^{T}\mathbf{Y}_{2} & \mathbf{X}^{T}\mathbf{Y}_{3} & \cdots& \mathbf{X}^{T}\mathbf{Y}_{m+1} \\ \mathbf{Y}_{2}^{T}\mathbf{X} & \mathbf{Y}_{2}^{T}\mathbf{Y}_{2} & \mathbf{Y}_{2}^{T}\mathbf{Y}_{3} & \cdots& \mathbf{Y}_{2}^{T}\mathbf{Y}_{m+1} \\ \mathbf{Y}_{3}^{T}\mathbf{X} & \mathbf{Y}_{3}^{T}\mathbf{Y}_{2} & \mathbf{Y}_{3}^{T}\mathbf{Y}_{3} & \cdots& \mathbf{Y}_{3}^{T}\mathbf{Y}_{m+1} \\ \vdots& \vdots& \vdots& \ddots& \vdots\\ \mathbf{Y}_{m+1}^{T}\mathbf{X} & \mathbf{Y}_{m+1}^{T}\mathbf{Y}_{2} & \mathbf{Y}_{m+1}^{T}\mathbf{Y}_{3} & \cdots& \mathbf{Y}_{m+1}^{T}\mathbf{Y}_{m+1} \end{matrix}\right)^{-1}. \end{matrix}$$

Each element in the matrices are the estimated effect sizes and the variance of the effect sizes are estimated using our proposed method.

Supplementary Table 1: Simulation results when the adjustment trait explains 2% of the variance in the outcome variable.

| Allele frequency | Individual level data (Gold standard) | | Proposed method  Full dataset | | Proposed method  Subset dataset (20%) | | Proposed method within *±*20% of true value | |
| --- | --- | --- | --- | --- | --- | --- | --- | --- |
|  | $\hat{\beta}$ | SE($\hat{\beta}$) | $\hat{\beta}$ | SE($\hat{\beta}$) | $\hat{\beta}$ | SE($\hat{\beta}$) | $\hat{\beta}$ | SE($\hat{\beta}$) |
| Continuous $\mathbf{Y}_{1}$ & continuous$\mathbf{Y}_{2}$ | | | | | | | | |
| 2% | 0.709 | 0.162 | 0.709 | 0.162 | 0.709 | 0.161 | 0.710 | 0.162 |
| 5% | 0.460 | 0.102 | 0.460 | 0.102 | 0.461 | 0.102 | 0.460 | 0.102 |
| 10% | 0.332 | 0.072 | 0.332 | 0.072 | 0.331 | 0.072 | 0.332 | 0.072 |
| 25% | 0.231 | 0.051 | 0.231 | 0.051 | 0.230 | 0.051 | 0.231 | 0.051 |
| Continuous $\mathbf{Y}_{1}$ & binary $\mathbf{Y}_{2}$ | | | | | | | | |
| 2% | 0.708 | 0.159 | 0.709 | 0.158 | 0.709 | 0.158 | 0.709 | 0.159 |
| 5% | 0.459 | 0.101 | 0.460 | 0.101 | 0.460 | 0.100 | 0.459 | 0.101 |
| 10% | 0.333 | 0.071 | 0.331 | 0.071 | 0.332 | 0.071 | 0.331 | 0.071 |
| 25% | 0.226 | 0.052 | 0.228 | 0.051 | 0.227 | 0.051 | 0.226 | 0.052 |

Number in the table represent averages over all simulation replicates.

Supplementary Table 2: Simulation results when the correlations are up to 20% underestimated or overestimated of the true value.

| Allele frequency | Individual level data frequency (Gold standard) | | Proposed method using up to 20% overestimate of true value | | Proposed method using up to 20% underestimate of true value | |
| --- | --- | --- | --- | --- | --- | --- |
|  | $\hat{\beta}$ | SE($\hat{\beta}$) | $\hat{\beta}$ | SE($\hat{\beta}$) | $\hat{\beta}$ | SE($\hat{\beta}$) |
| Continuous $\mathbf{Y}_{1}$ & continuous $\mathbf{Y}_{2}$ | | | | | | |
| 2% | 0.71 | 0.146 | 0.659 | 0.141 | 0.761 | 0.15 |
| 5% | 0.46 | 0.092 | 0.43 | 0.09 | 0.493 | 0.095 |
| 10% | 0.332 | 0.065 | 0.309 | 0.063 | 0.356 | 0.067 |
| 25% | 0.231 | 0.046 | 0.215 | 0.045 | 0.247 | 0.048 |
| Continuous $\mathbf{Y}_{1}$ & binary $\mathbf{Y}_{2}$ | | | | | | |
| 2% | 0.709 | 0.143 | 0.692 | 0.139 | 0.722 | 0.147 |
| 5% | 0.46 | 0.091 | 0.44 | 0.088 | 0.471 | 0.093 |
| 10% | 0.333 | 0.064 | 0.312 | 0.062 | 0.346 | 0.066 |
| 25% | 0.228 | 0.046 | 0.204 | 0.045 | 0.24 | 0.048 |
| Binary $\mathbf{Y}_{1}$ & continuous $\mathbf{Y}_{2}$ | | | | | | |
| 2% | 0.597 | 1.072 | 0.513 | 1.049 | 0.599 | 1.047 |
| 5% | 0.752 | 0.288 | 0.664 | 0.273 | 0.721 | 0.272 |
| 10% | 0.809 | 0.203 | 0.728 | 0.193 | 0.772 | 0.192 |
| 25% | 0.862 | 0.15 | 0.791 | 0.143 | 0.823 | 0.143 |
| Binary $\mathbf{Y}_{1}$ & binary $\mathbf{Y}_{2}$ | | | | | | |
| 2% | 0.867 | 0.364 | 0.765 | 0.34 | 0.805 | 0.34 |
| 5% | 0.886 | 0.234 | 0.786 | 0.219 | 0.827 | 0.22 |
| 10% | 0.842 | 0.172 | 0.76 | 0.162 | 0.803 | 0.163 |
| 25% | 0.764 | 0.133 | 0.72 | 0.129 | 0.767 | 0.13 |

Supplementary Table 3: Simulation results for type I error when the genetic variant is not associated with outcome or covariate (α=0.05)

|  | Allele frequency | Gold standard | Proposed method Full dataset | Proposed method Subset dataset (20%) | Proposed method within ±20% of true value |
| --- | --- | --- | --- | --- | --- |
|  |  |  |  |  |  |
|  |  |  |  |  |  |
| Continuous $\mathbf{Y}_{1}$ & continuous$\mathbf{Y}_{2}$ | 2% | 0.056 | 0.056 | 0.058 | 0.051 |
|  | 5% | 0.047 | 0.047 | 0.048 | 0.046 |
|  | 10% | 0.059 | 0.059 | 0.056 | 0.06 |
|  | 25% | 0.051 | 0.051 | 0.052 | 0.05 |
| Continuous $\mathbf{Y}_{1}$ & binary $\mathbf{Y}_{2}$ | 2% | 0.059 | 0.059 | 0.061 | 0.059 |
|  | 5% | 0.041 | 0.041 | 0.043 | 0.042 |
|  | 10% | 0.063 | 0.063 | 0.058 | 0.061 |
|  | 25% | 0.052 | 0.052 | 0.052 | 0.053 |
| Binary $\mathbf{Y}_{1}$ & continuous $\mathbf{Y}_{2}$ | 2% | 0.044 | 0.035 | 0.033 | 0.031 |
|  | 5% | 0.05 | 0.041 | 0.044 | 0.041 |
|  | 10% | 0.04 | 0.031 | 0.033 | 0.034 |
|  | 25% | 0.059 | 0.04 | 0.041 | 0.043 |
| Binary $\mathbf{Y}_{1}$ & binary $\mathbf{Y}_{2}$ | 2% | 0.05 | 0.05 | 0.053 | 0.052 |
|  | 5% | 0.047 | 0.046 | 0.043 | 0.048 |
|  | 10% | 0.042 | 0.042 | 0.046 | 0.044 |
|  | 25% | 0.046 | 0.046 | 0.046 | 0.046 |

Supplementary Table 4: Simulation results for type I error when the genetic variant is not associated with outcome but associated with the covariate (α=0.05)

|  | Allele frequency | Gold standard | Proposed method Full dataset | Proposed method Subset dataset (20%) | Proposed method within ±20% of true value |
| --- | --- | --- | --- | --- | --- |
| Continuous $\mathbf{Y}_{1}$ & continuous $\mathbf{Y}_{2}$ | 2% | 0.064 | 0.063 | 0.084 | 0.09 |
|  | 5% | 0.049 | 0.049 | 0.065 | 0.068 |
|  | 10% | 0.051 | 0.051 | 0.074 | 0.079 |
|  | 25% | 0.052 | 0.052 | 0.063 | 0.08 |
| Continuous $\mathbf{Y}_{1}$ & binary $\mathbf{Y}_{2}$ | 2% | 0.062 | 0.062 | 0.057 | 0.059 |
|  | 5% | 0.045 | 0.046 | 0.058 | 0.043 |
|  | 10% | 0.057 | 0.057 | 0.063 | 0.064 |
|  | 25% | 0.057 | 0.06 | 0.071 | 0.062 |
| Binary $\mathbf{Y}_{1}$ & continuous $\mathbf{Y}_{2}$ | 2% | 0.053 | 0.047 | 0.063 | 0.052 |
|  | 5% | 0.04 | 0.034 | 0.054 | 0.055 |
|  | 10% | 0.049 | 0.044 | 0.051 | 0.046 |
|  | 25% | 0.061 | 0.057 | 0.057 | 0.067 |
| Binary $\mathbf{Y}_{1}$ & binary $\mathbf{Y}_{2}$ | 2% | 0.053 | 0.053 | 0.057 | 0.052 |
|  | 5% | 0.045 | 0.045 | 0.046 | 0.044 |
|  | 10% | 0.044 | 0.044 | 0.046 | 0.043 |
|  | 25% | 0.045 | 0.045 | 0.041 | 0.046 |

Supplementary Table 5: Simulation results for power when the allele frequency =25%

|  | Variance explained by $\mathbf{Y}_{2}$ | Gold standard | Proposed method Full dataset | Proposed method Subset dataset (20%) | Proposed method within ±20% of true value |
| --- | --- | --- | --- | --- | --- |
|  |  |  |  |  |  |
|  |  |  |  |  |  |
| Continuous $\mathbf{Y}_{1}$ & continuous $\mathbf{Y}_{2}$ | 5% | 0.908 | 0.908 | 0.904 | 0.91 |
|  | 10% | 0.92 | 0.92 | 0.918 | 0.923 |
|  | 20% | 0.951 | 0.951 | 0.945 | 0.949 |
|  | 40% | 0.986 | 0.986 | 0.987 | 0.982 |
| Continuous $\mathbf{Y}_{1}$ & binary $\mathbf{Y}_{2}$ | 5% | 0.9 | 0.9 | 0.899 | 0.901 |
|  | 10% | 0.915 | 0.915 | 0.912 | 0.914 |
|  | 20% | 0.939 | 0.938 | 0.938 | 0.941 |
|  | 40% | 0.98 | 0.982 | 0.98 | 0.982 |
| Binary $\mathbf{Y}_{1}$ & continuous $\mathbf{Y}_{2}$ | 5% | 0.785 | 0.784 | 0.783 | 0.785 |
|  | 10% | 0.815 | 0.812 | 0.806 | 0.81 |
|  | 20% | 0.862 | 0.857 | 0.853 | 0.856 |
|  | 40% | 0.938 | 0.93 | 0.93 | 0.931 |
| Binary $\mathbf{Y}_{1}$ & binary $\mathbf{Y}_{2}$ | 5% | 0.786 | 0.781 | 0.78 | 0.776 |
|  | 10% | 0.8 | 0.799 | 0.795 | 0.794 |
|  | 20% | 0.85 | 0.839 | 0.841 | 0.842 |
|  | 40% | 0.935 | 0.923 | 0.923 | 0.925 |

Supplementary Table 6: Simulation results for power when the allele frequency =10%

|  | Variance explained by $\mathbf{Y}_{2}$ | Gold standard | Proposed method Full dataset | Proposed method Subset dataset (20%) | Proposed method within ±20% of true value |
| --- | --- | --- | --- | --- | --- |
|  |  |  |  |  |  |
|  |  |  |  |  |  |
| Continuous $\mathbf{Y}_{1}$ & continuous $\mathbf{Y}_{2}$ | 5% | 0.912 | 0.912 | 0.906 | 0.913 |
|  | 10% | 0.924 | 0.924 | 0.92 | 0.923 |
|  | 20% | 0.949 | 0.949 | 0.951 | 0.95 |
|  | 40% | 0.989 | 0.989 | 0.988 | 0.983 |
| Continuous $\mathbf{Y}_{1}$ & binary $\mathbf{Y}_{2}$ | 5% | 0.917 | 0.917 | 0.918 | 0.919 |
|  | 10% | 0.925 | 0.928 | 0.927 | 0.926 |
|  | 20% | 0.951 | 0.951 | 0.949 | 0.952 |
|  | 40% | 0.987 | 0.988 | 0.989 | 0.986 |
| Binary $\mathbf{Y}_{1}$ & continuous $\mathbf{Y}_{2}$ | 5% | 0.822 | 0.82 | 0.821 | 0.818 |
|  | 10% | 0.838 | 0.836 | 0.834 | 0.836 |
|  | 20% | 0.88 | 0.874 | 0.871 | 0.871 |
|  | 40% | 0.958 | 0.953 | 0.95 | 0.953 |
| Binary $\mathbf{Y}_{1}$ & binary $\mathbf{Y}_{2}$ | 5% | 0.811 | 0.809 | 0.806 | 0.81 |
|  | 10% | 0.833 | 0.828 | 0.831 | 0.83 |
|  | 20% | 0.873 | 0.865 | 0.867 | 0.866 |
|  | 40% | 0.956 | 0.941 | 0.941 | 0.942 |

Supplementary Table 7: Simulation results for power estimates when the allele frequency =5%

|  | Variance explained by $\mathbf{Y}_{2}$ | Gold standard | Proposed method Full dataset | Proposed method Subset dataset (20%) | Proposed method within ±20% of true value |
| --- | --- | --- | --- | --- | --- |
|  |  |  |  |  |  |
|  |  |  |  |  |  |
| Continuous $\mathbf{Y}_{1}$ & continuous $\mathbf{Y}_{2}$ | 5% | 0.905 | 0.904 | 0.903 | 0.905 |
|  | 10% | 0.924 | 0.924 | 0.922 | 0.921 |
|  | 20% | 0.944 | 0.944 | 0.944 | 0.942 |
|  | 40% | 0.984 | 0.984 | 0.985 | 0.987 |
| Continuous $\mathbf{Y}_{1}$ & binary $\mathbf{Y}_{2}$ | 5% | 0.898 | 0.898 | 0.896 | 0.898 |
|  | 10% | 0.915 | 0.914 | 0.916 | 0.918 |
|  | 20% | 0.948 | 0.947 | 0.944 | 0.946 |
|  | 40% | 0.988 | 0.988 | 0.988 | 0.988 |
| Binary $\mathbf{Y}_{1}$ & continuous $\mathbf{Y}_{2}$ | 5% | 0.848 | 0.847 | 0.844 | 0.851 |
|  | 10% | 0.863 | 0.861 | 0.853 | 0.859 |
|  | 20% | 0.886 | 0.882 | 0.885 | 0.882 |
|  | 40% | 0.957 | 0.954 | 0.952 | 0.952 |
| Binary $\mathbf{Y}_{1}$ & binary $\mathbf{Y}_{2}$ | 5% | 0.817 | 0.816 | 0.816 | 0.816 |
|  | 10% | 0.831 | 0.829 | 0.829 | 0.827 |
|  | 20% | 0.868 | 0.863 | 0.864 | 0.862 |
|  | 40% | 0.954 | 0.942 | 0.94 | 0.943 |

Supplementary Table 8: Simulation results for power when the allele frequency =2%

|  | Variance explained by $\mathbf{Y}_{2}$ | Gold standard | Proposed method Full dataset | Proposed method Subset dataset (20%) | Proposed method within ±20% of true value |
| --- | --- | --- | --- | --- | --- |
|  |  |  |  |  |  |
|  |  |  |  |  |  |
| Continuous $\mathbf{Y}_{1}$ & continuous $\mathbf{Y}_{2}$ | 5% | 0.888 | 0.888 | 0.886 | 0.887 |
|  | 10% | 0.898 | 0.898 | 0.9 | 0.901 |
|  | 20% | 0.929 | 0.929 | 0.927 | 0.928 |
|  | 40% | 0.979 | 0.979 | 0.98 | 0.979 |
| Continuous $\mathbf{Y}_{1}$ & binary $\mathbf{Y}_{2}$ | 5% | 0.897 | 0.897 | 0.897 | 0.898 |
|  | 10% | 0.909 | 0.909 | 0.911 | 0.908 |
|  | 20% | 0.932 | 0.929 | 0.932 | 0.929 |
|  | 40% | 0.974 | 0.972 | 0.97 | 0.972 |
| Binary $\mathbf{Y}_{1}$ & continuous $\mathbf{Y}_{2}$ | 5% | 0.841 | 0.838 | 0.833 | 0.838 |
|  | 10% | 0.859 | 0.853 | 0.852 | 0.854 |
|  | 20% | 0.89 | 0.885 | 0.885 | 0.888 |
|  | 40% | 0.952 | 0.948 | 0.942 | 0.948 |
| Binary $\mathbf{Y}_{1}$ & binary $\mathbf{Y}_{2}$ | 5% | 0.835 | 0.832 | 0.829 | 0.833 |
|  | 10% | 0.857 | 0.851 | 0.848 | 0.847 |
|  | 20% | 0.888 | 0.886 | 0.884 | 0.885 |
|  | 40% | 0.947 | 0.939 | 0.939 | 0.939 |

Supplementary Table 9: Type I error under different ratio of subset sample size to full data sample size (α=0.05)

| Allele frequency | Ratio | | | | | | |
| --- | --- | --- | --- | --- | --- | --- | --- |
|  | 0.3 | 0.4 | 0.5 | 0.6 | 0.7 | 0.8 | 0.9 |
| 2% | 0.085 | 0.084 | 0.082 | 0.075 | 0.074 | 0.069 | 0.065 |
| 5% | 0.061 | 0.063 | 0.063 | 0.065 | 0.061 | 0.055 | 0.05 |
| 10% | 0.061 | 0.064 | 0.056 | 0.059 | 0.058 | 0.053 | 0.05 |
| 25% | 0.064 | 0.064 | 0.062 | 0.063 | 0.058 | 0.057 | 0.053 |

Supplementary Table 10: Type I error of random estimates within certain boundaries of the true correlation between the outcome and the covariate (α=0.05)

| Allele frequency | Proportion of the true estimates (*k*) | | |
| --- | --- | --- | --- |
|  | 0.15 | 0.1 | 0.05 |
| 2% | 0.082 | 0.073 | 0.064 |
| 5% | 0.064 | 0.055 | 0.053 |
| 10% | 0.074 | 0.07 | 0.071 |
| 25% | 0.065 | 0.061 | 0.058 |

Generating $\hat{\gamma}_{m}$ from uniform((1- *k*)*$\hat{\gamma}_{m}^{*}$, (1+ *k*)*$\hat{\gamma}_{m}^{*}$)


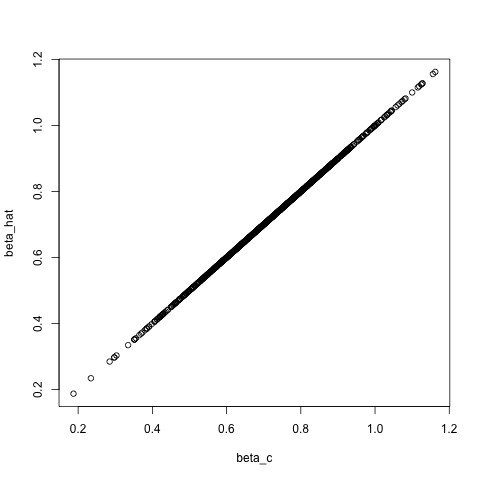

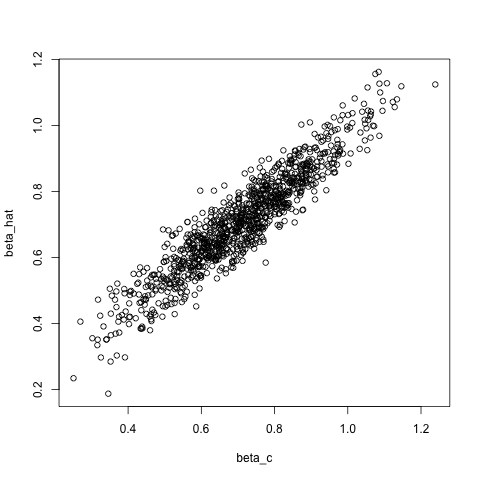

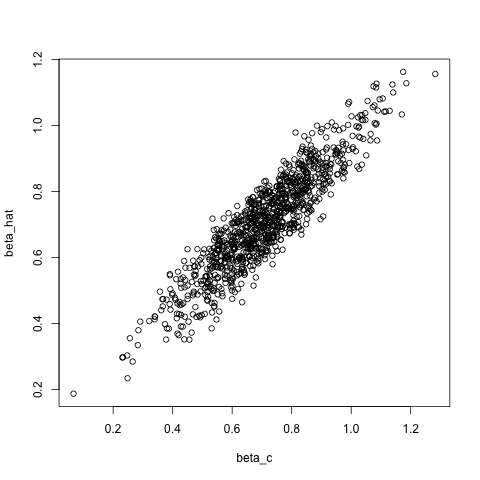


(a) full data, MAF=2% (b) subset data, MAF=2% (c) random, MAF=2%


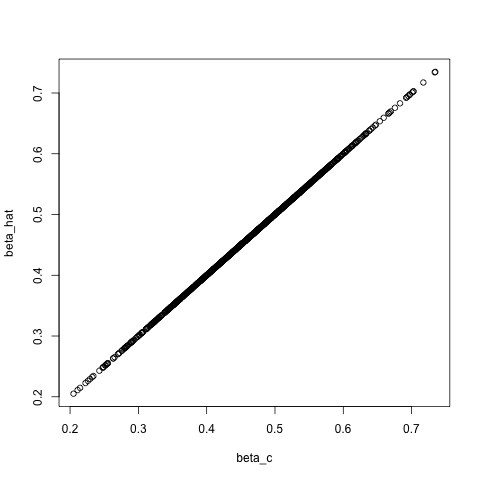

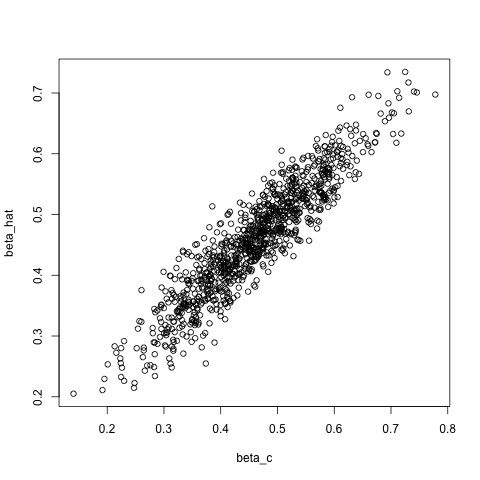

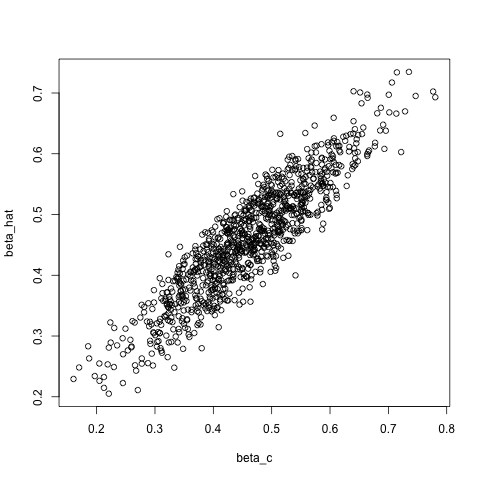


(d) full data, MAF=5% (e) subset data, MAF=5% (f) random, MAF=5%


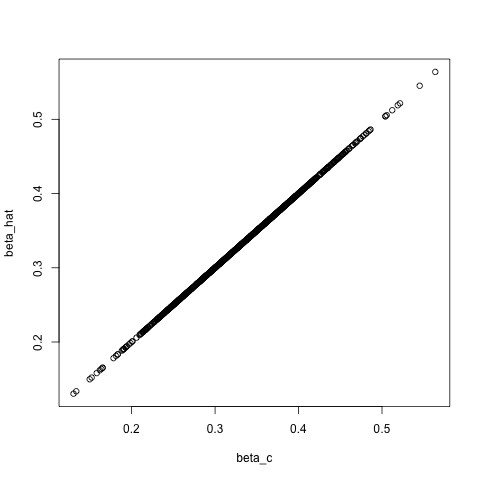

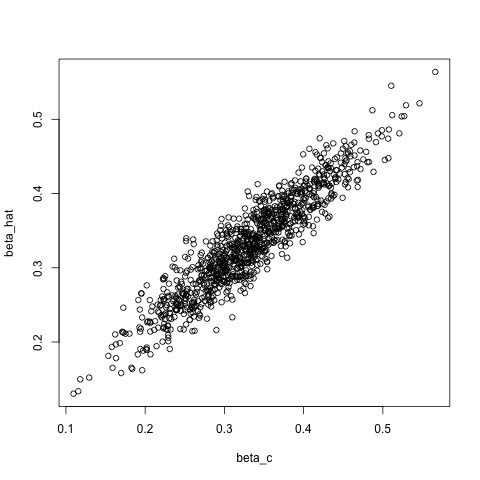

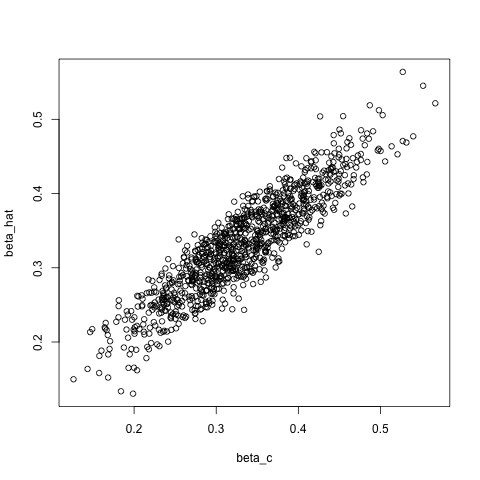


(g) full data, MAF=10% (h) subset data, MAF=10% (i) random, MAF=10%


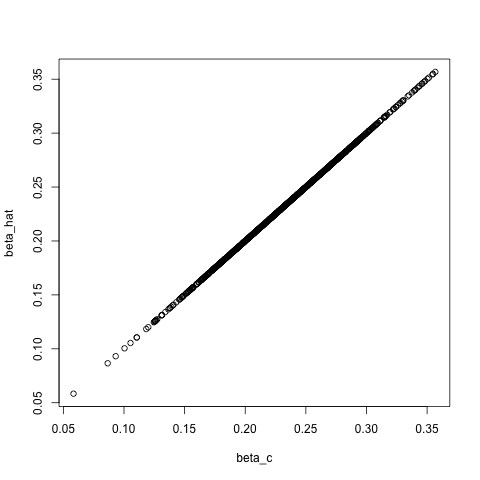

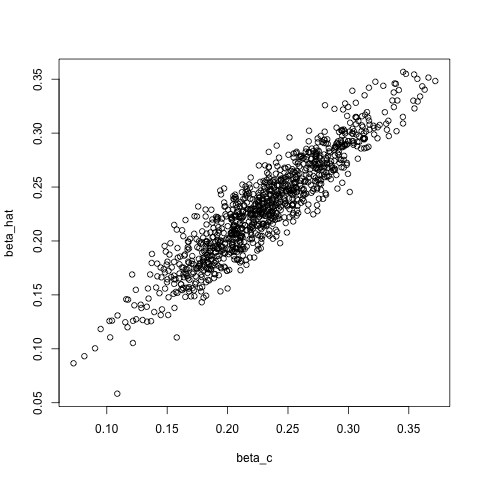

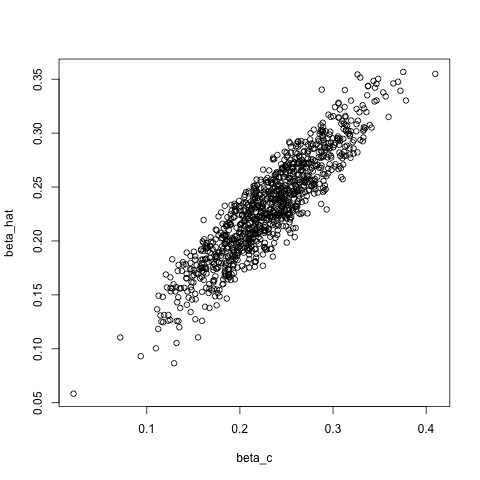


(j) full data, MAF=25% (k) subset data, MAF=25% (l) random, MAF=25%

Supplementary Figure 1: Beta estimates for two continuous traits. “beta c” means beta estimates calculated by our proposed method. “beta hat” means beta estimates calculated by individual level data (gold standard)


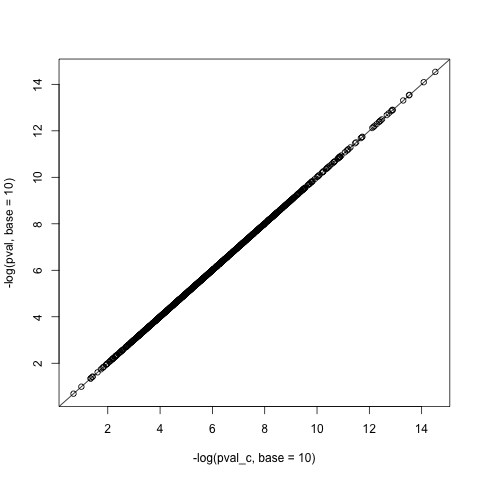

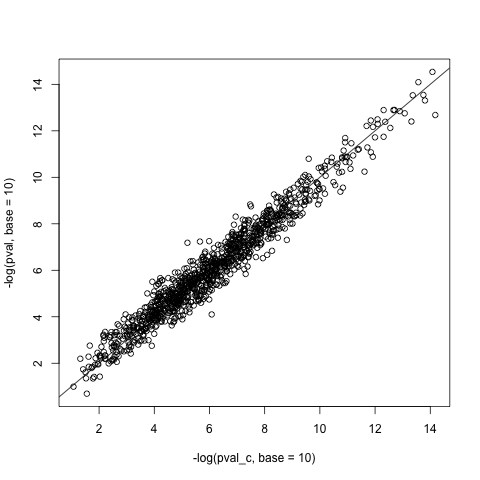

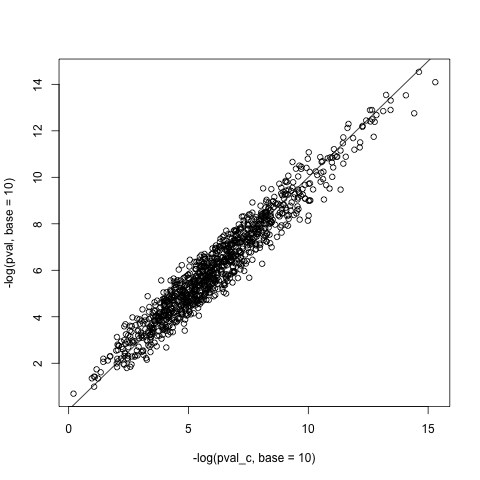


(a) full data, MAF=2% (b) subset data, MAF=2% (c) random, MAF=2%


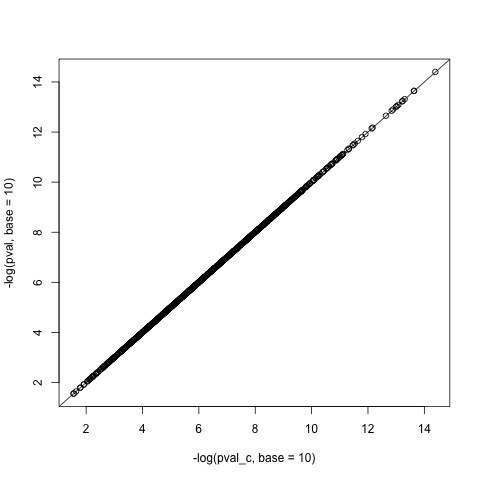

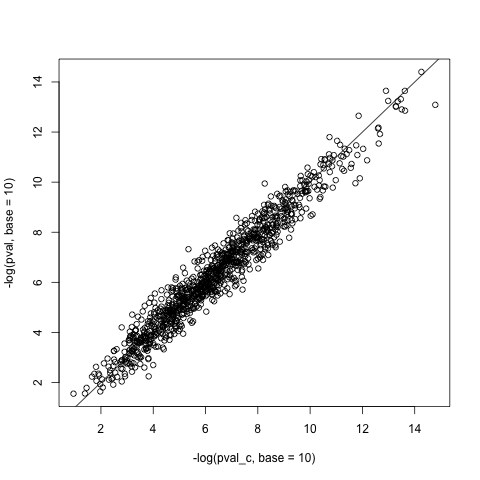

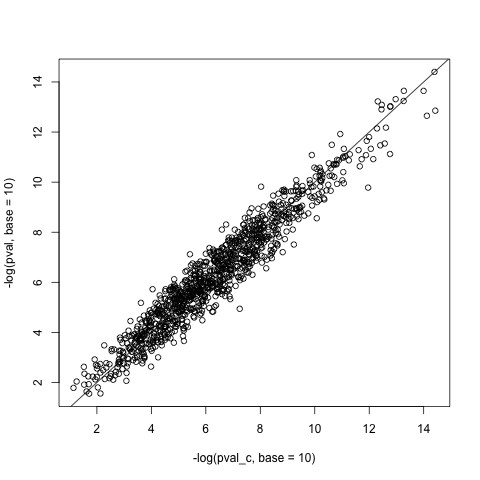


(d) full data, MAF=5% (e) subset data, MAF=5% (f) random, MAF=5%


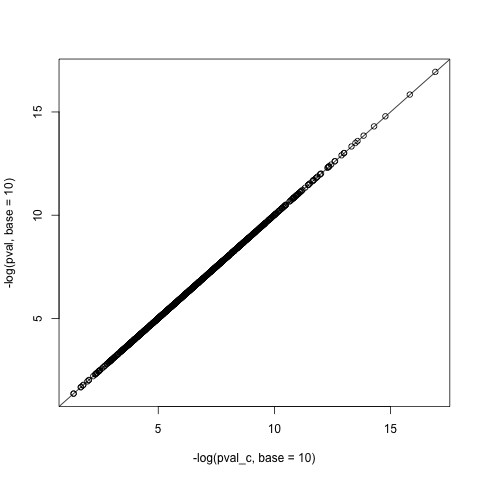

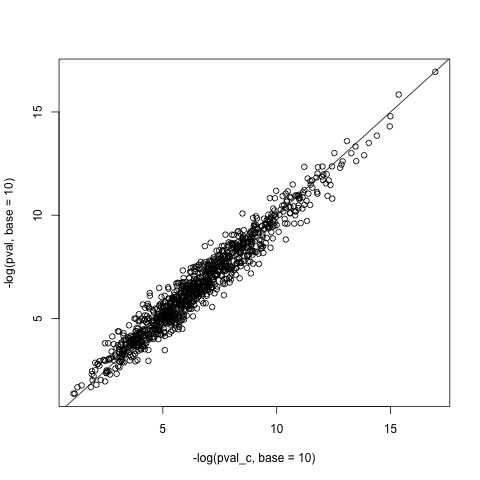

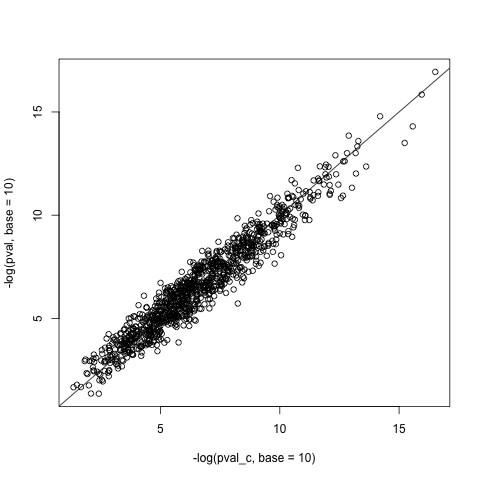


(g) full data, MAF=10% (h) subset data, MAF=10% (i) random, MAF=10%


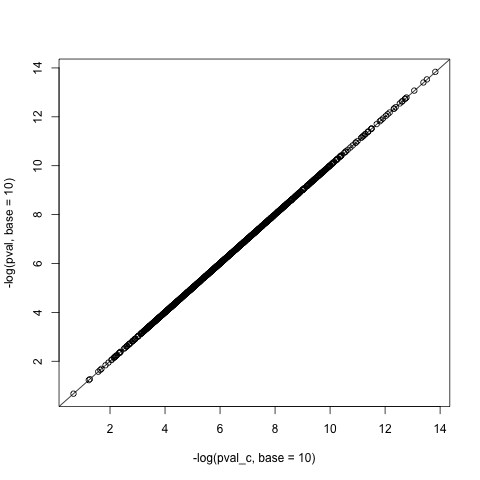

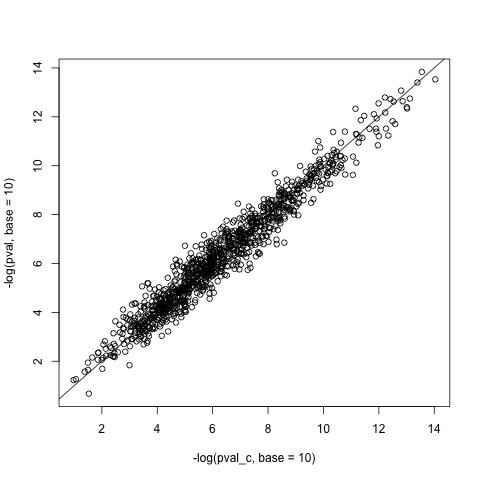

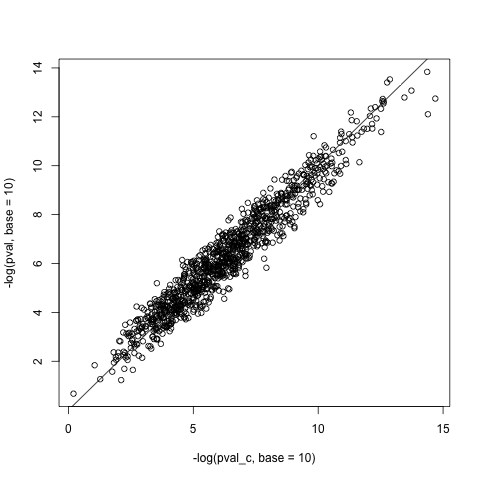


(j) full data, MAF=25% (k) subset data, MAF=25% (l) random, MAF=25%

Supplementary Figure 2: *p*-values for two continuous traits. “pval c” means *p*-values calculated by our proposed method. “beta hat” means *p*-values calculated by individual level data (gold standard)


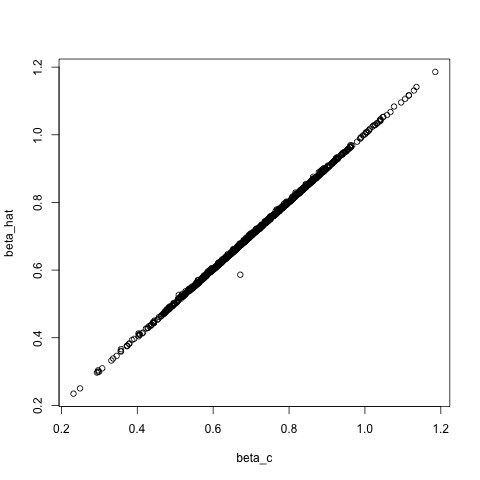

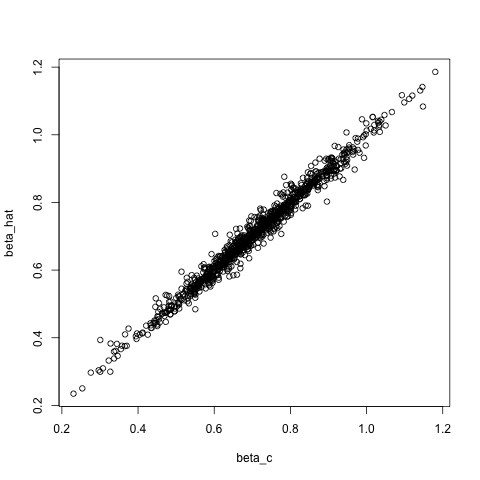

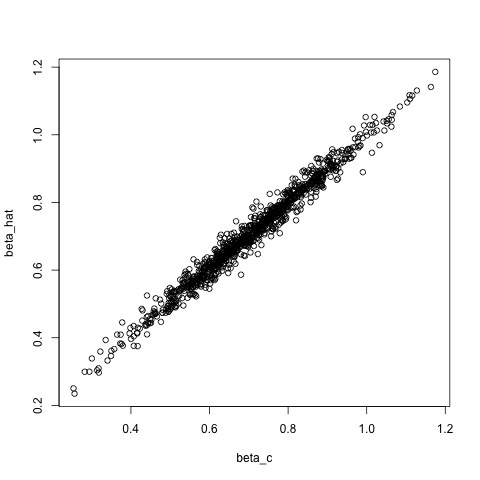


(a) full data, MAF=2% (b) subset data, MAF=2% (c) random, MAF=2%


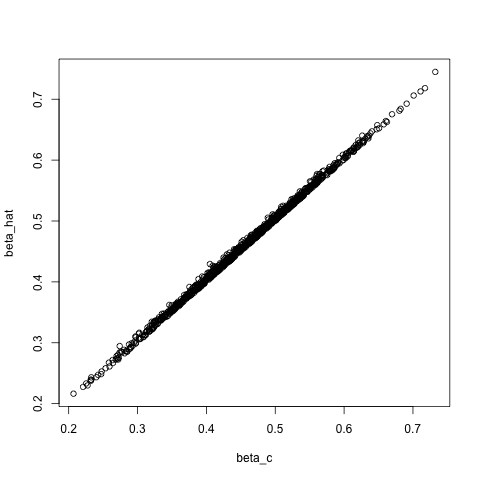

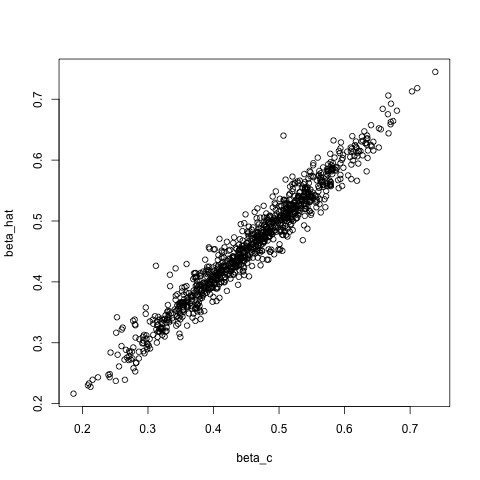

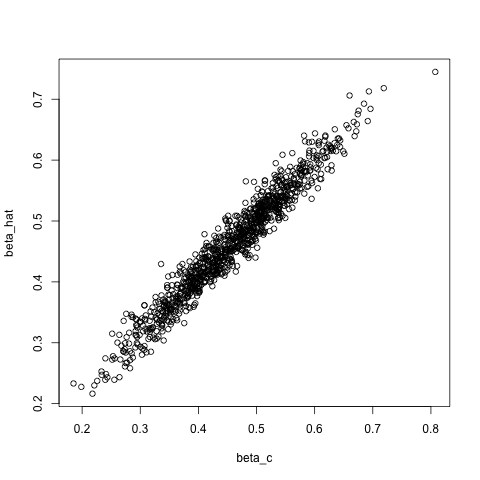


(d) full data, MAF=5% (e) subset data, MAF=5% (f) random, MAF=5%


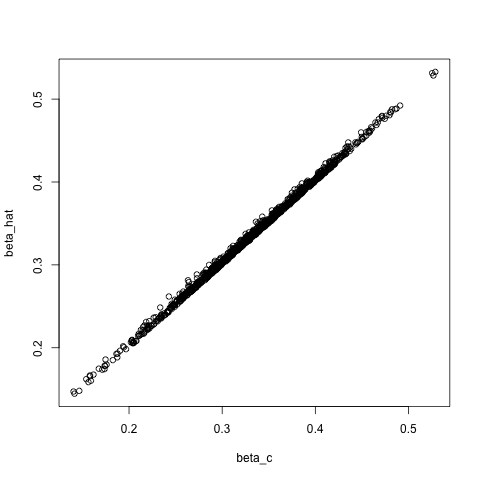

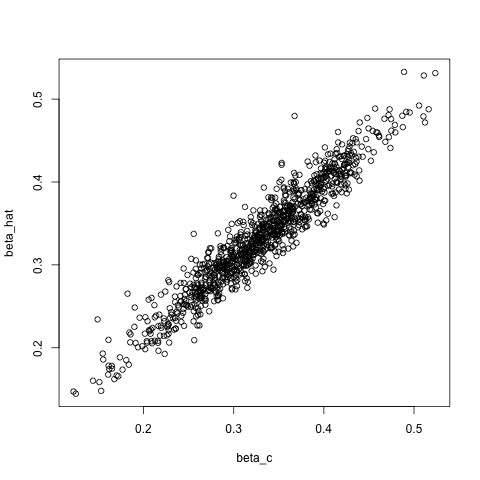

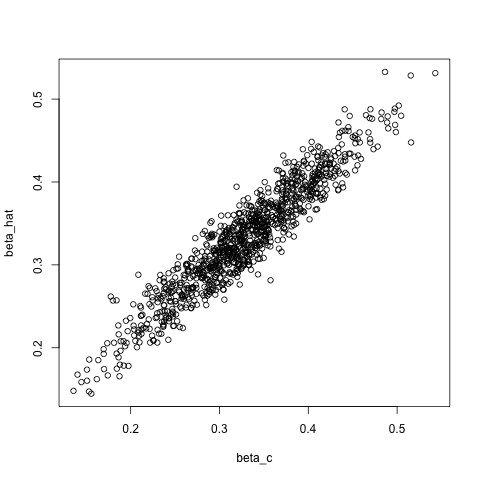


(g) full data, MAF=10% (h) subset data, MAF=10% (i) random, MAF=10%


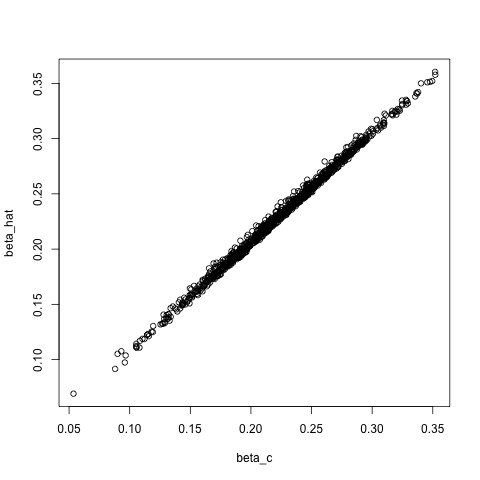

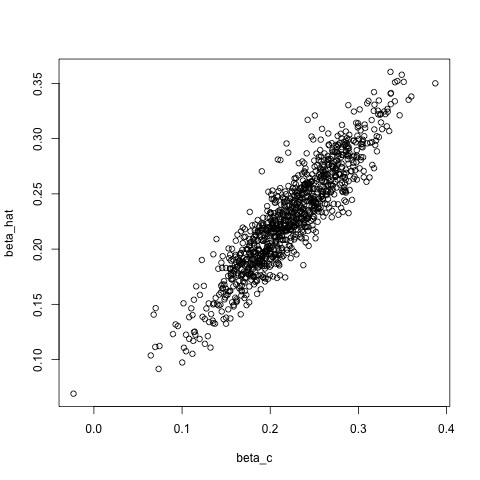

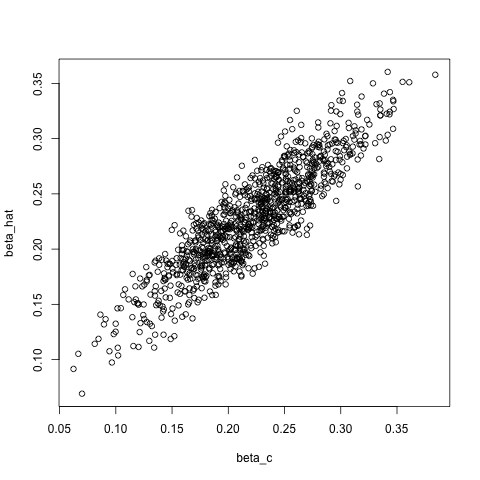


(j) full data, MAF=25% (k) subset data, MAF=25% (l) random, MAF=25%

Supplementary Figure 3: Beta estimates for continuous Y1 and binary Y2. “beta c” means beta estimates calculated by our proposed method. “beta hat” means beta estimates calculated by individual level data (gold standard)


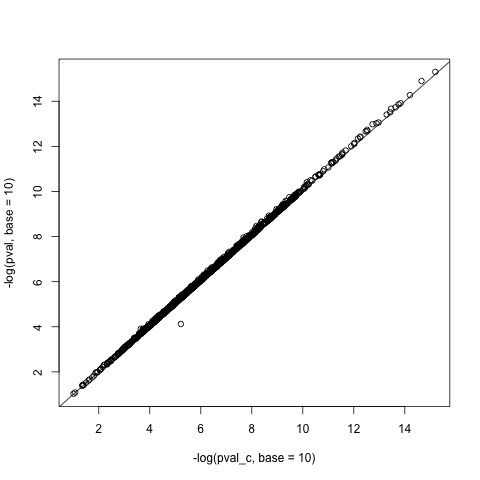

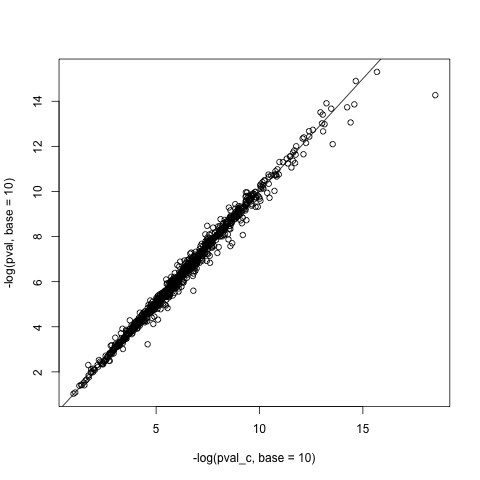

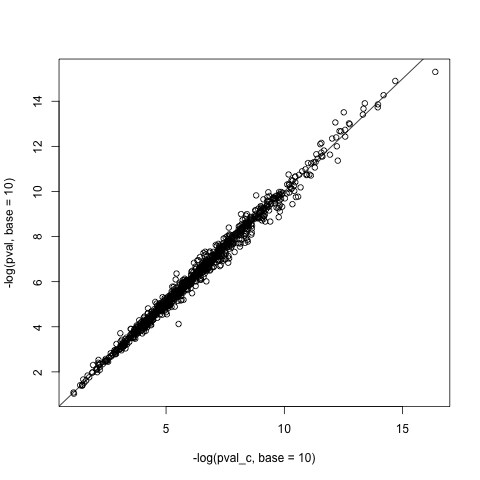


(a) full data, MAF=2% (b) subset data, MAF=2% (c) random, MAF=2%


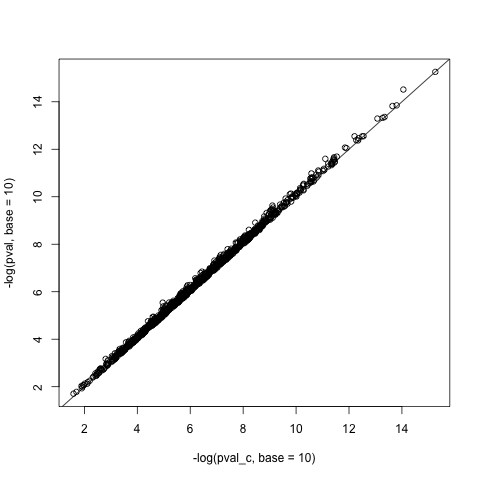

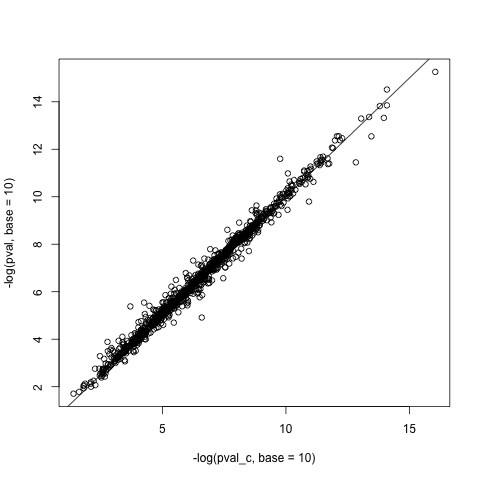

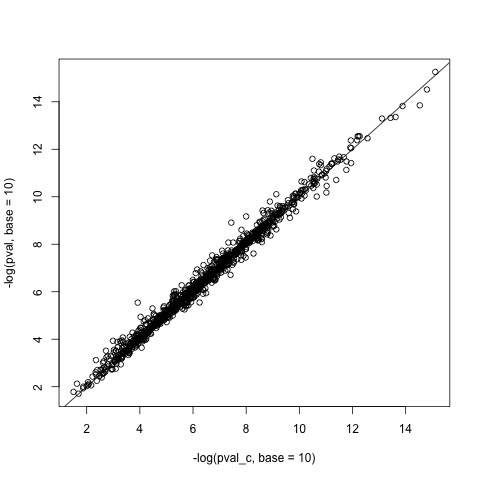


(d) full data, MAF=5% (e) subset data, MAF=5% (f) random, MAF=5%


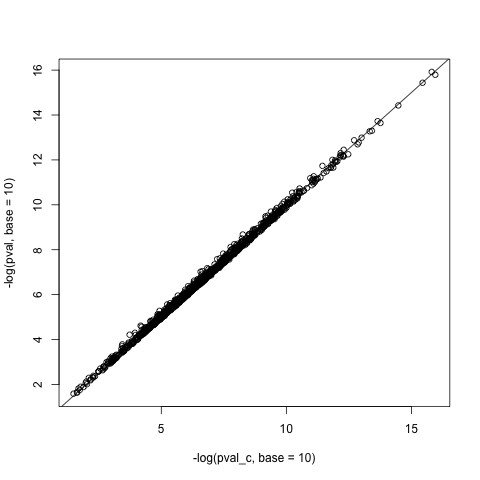

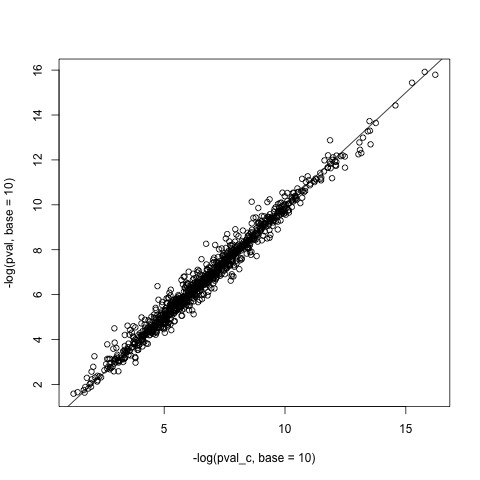

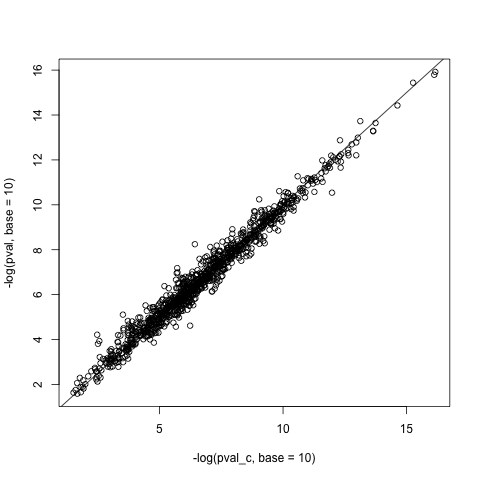


(g) full data, MAF=10% (h) subset data, MAF=10% (i) random, MAF=10%


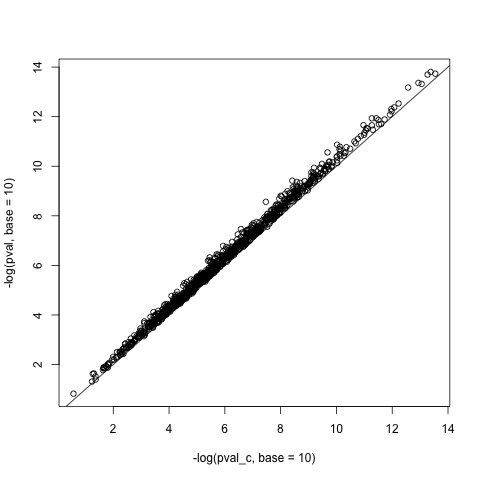

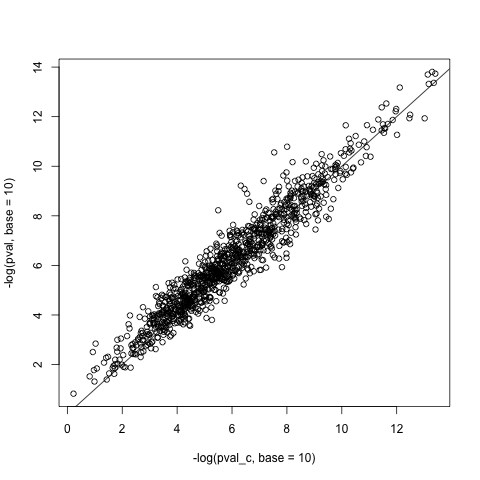

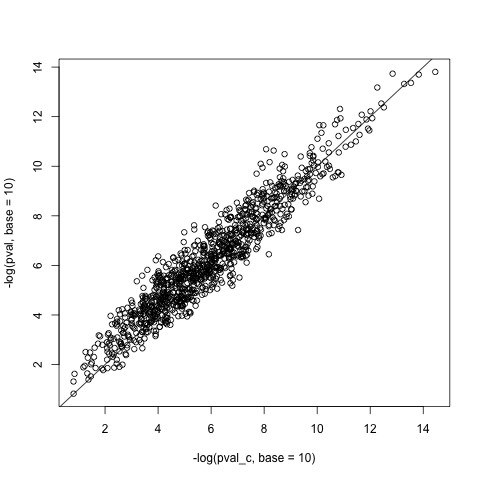


(j) full data, MAF=25% (k) subset data, MAF=25% (l) random, MAF=25%

Supplementary Figure 4: *p*-values for continuous Y1 and binary Y2. “pval c” means *p*-values calculated by our proposed method. “beta hat” means *p*-values calculated by individual level data (gold standard)


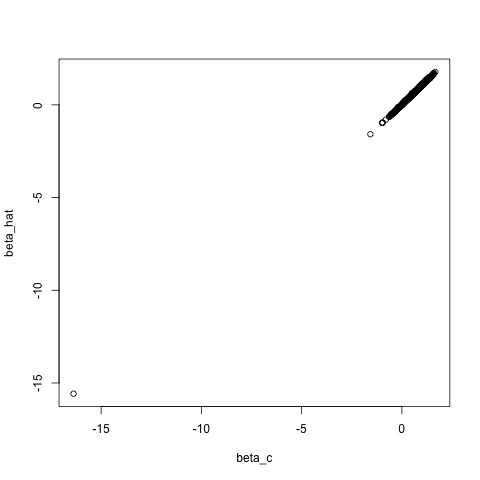

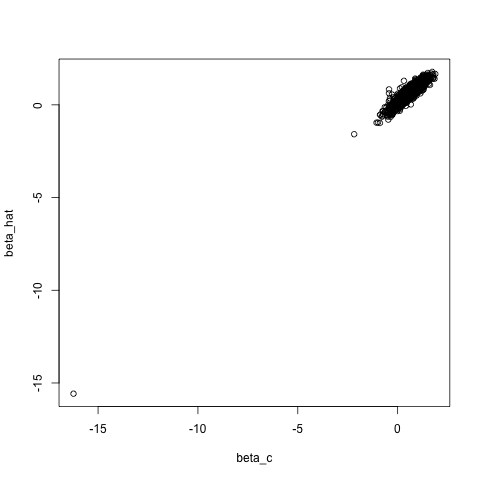

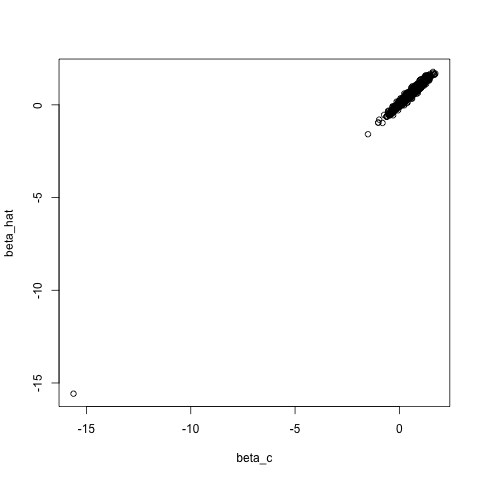


(a) full data, MAF=2% (b) subset data, MAF=2% (c) random, MAF=2%


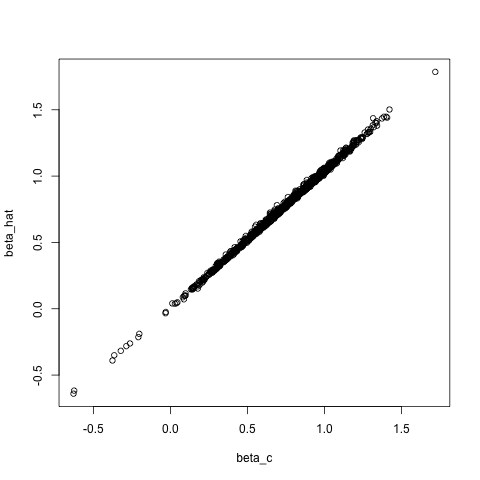

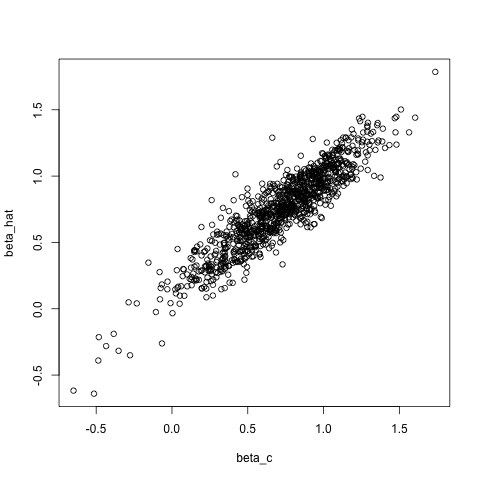

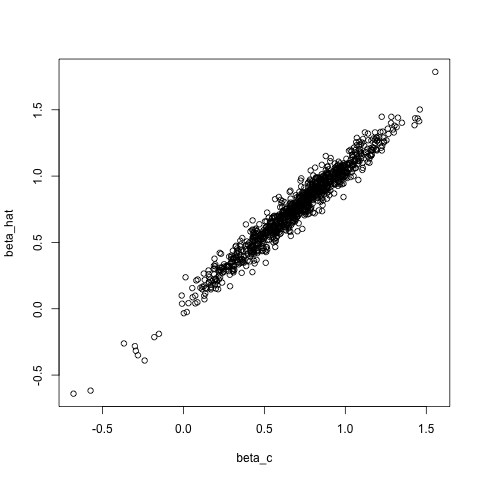


(d) full data, MAF=5% (e) subset data, MAF=5% (f) random, MAF=5%


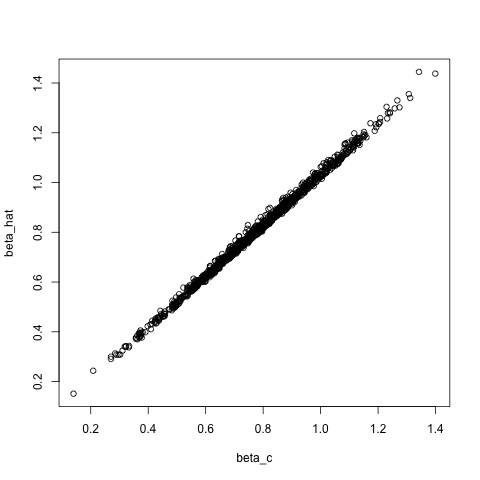

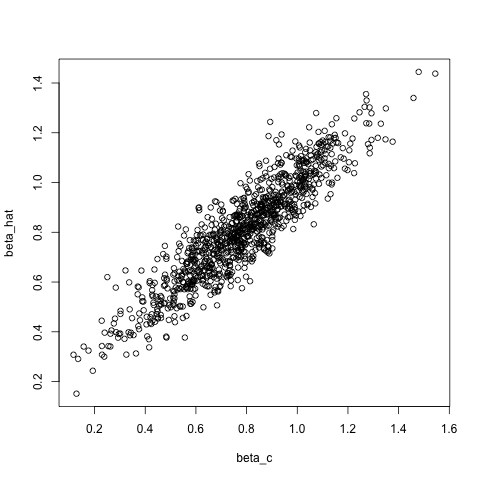

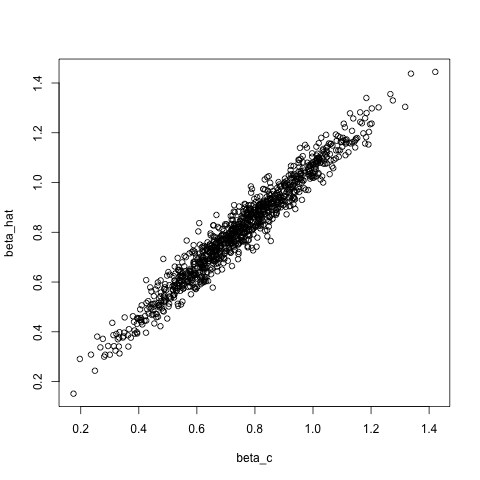


(g) full data, MAF=10% (h) subset data, MAF=10% (i) random, MAF=10%


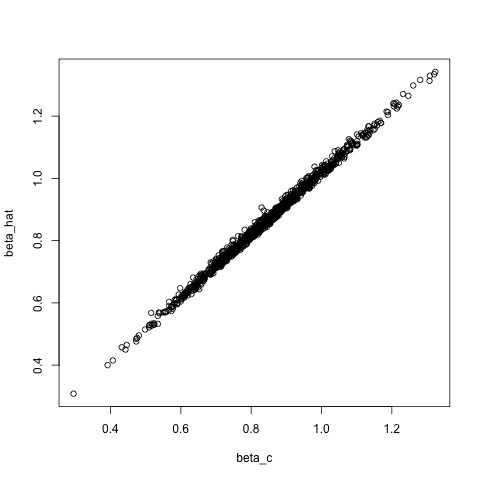

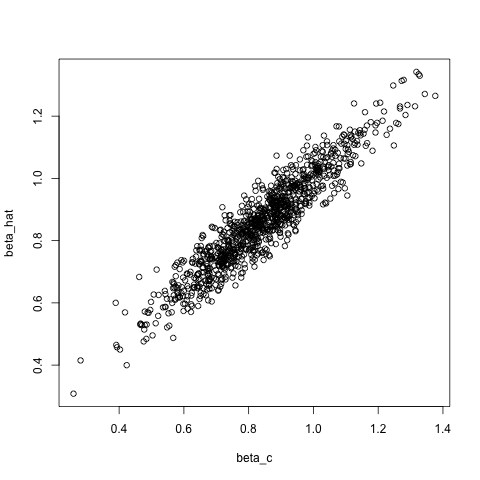

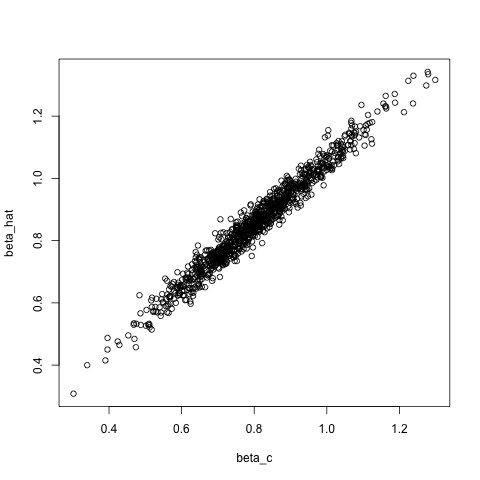


(j) full data, MAF=25% (k) subset data, MAF=25% (l) random, MAF=25%

Supplementary Figure 5: Beta estimates for binary Y1 and continuous Y2. “beta c” means beta estimates calculated by our proposed method. “beta hat” means beta estimates calculated by individual level data (gold standard)


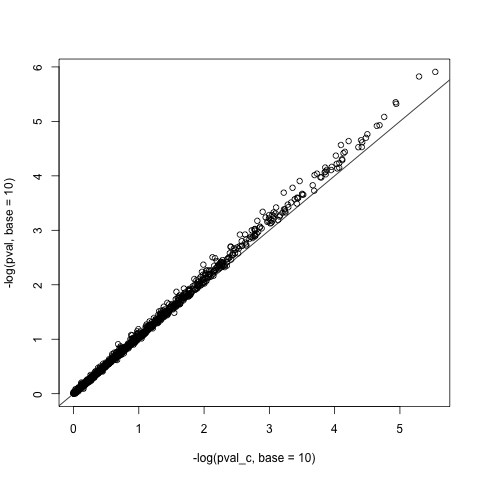

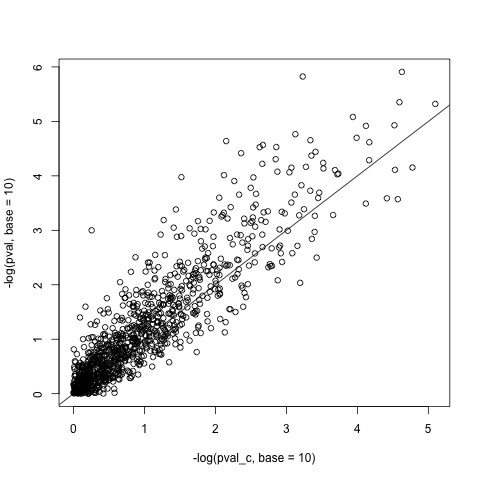

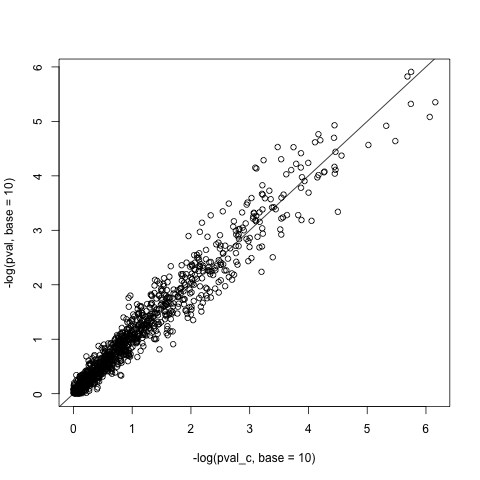


(a) full data, MAF=2% (b) subset data, MAF=2% (c) random, MAF=2%


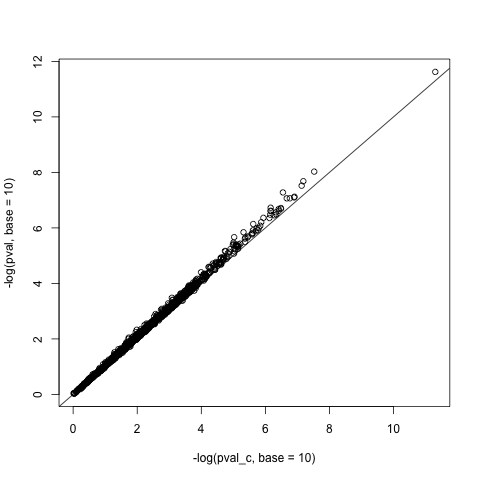

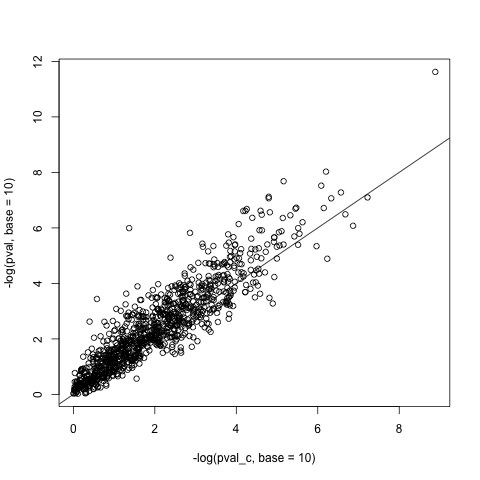

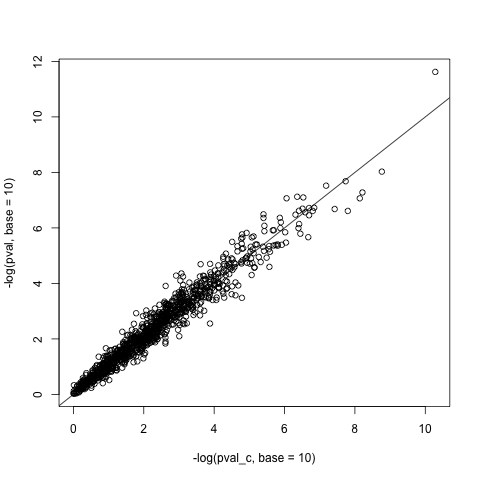


(d) full data, MAF=5% (e) subset data, MAF=5% (f) random, MAF=5%


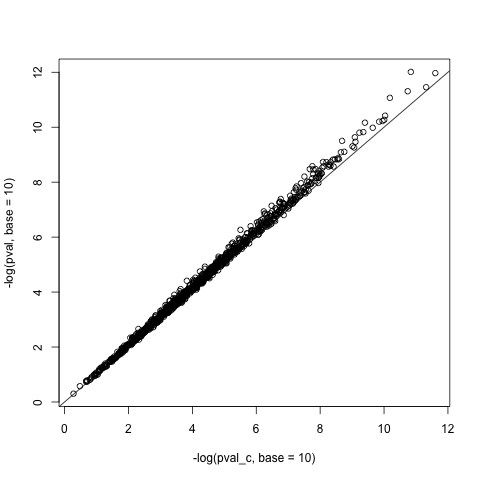

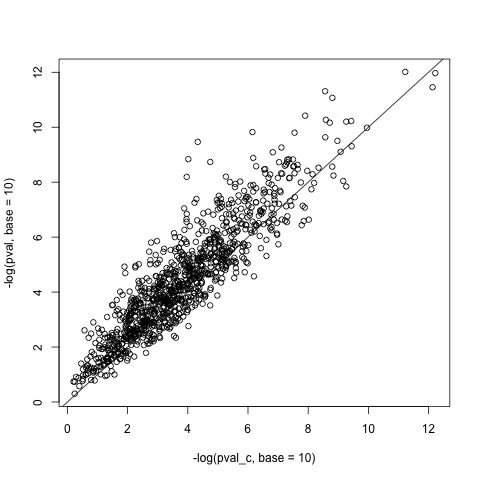

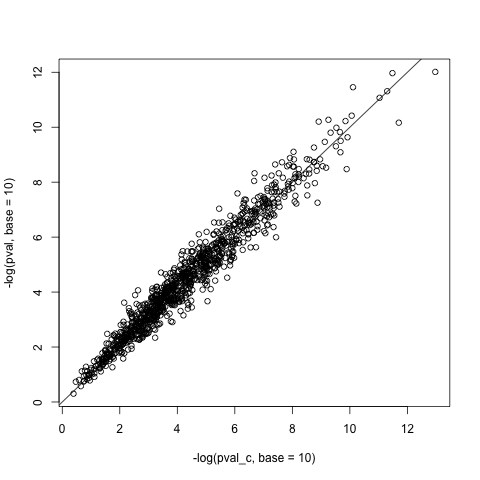


(g) full data, MAF=10% (h) subset data, MAF=10% (i) random, MAF=10%


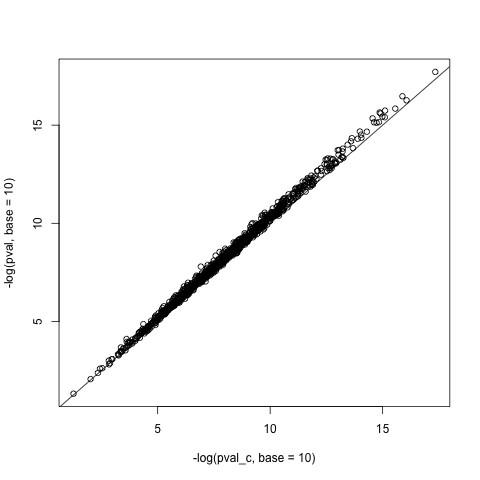

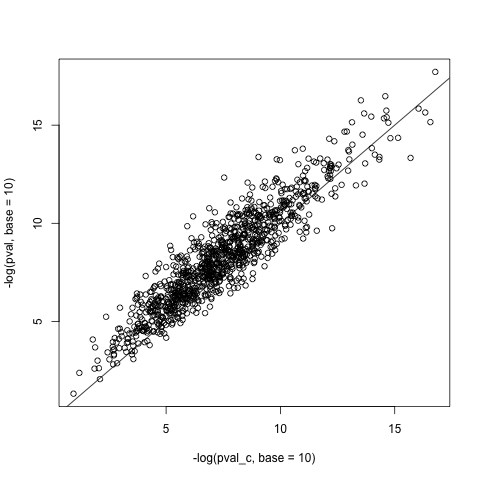

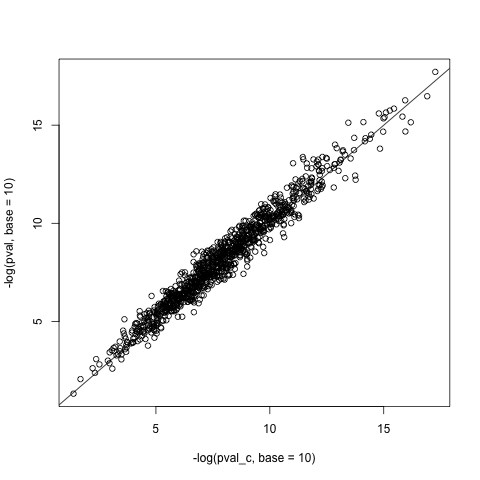


(j) full data, MAF=25% (k) subset data, MAF=25% (l) random, MAF=25%

Supplementary Figure 6: *p*-values for binary Y1 and continuous Y2. “pval c” means *p*-values calculated by our proposed method. “beta hat” means *p*-values calculated by individual level data (gold standard)


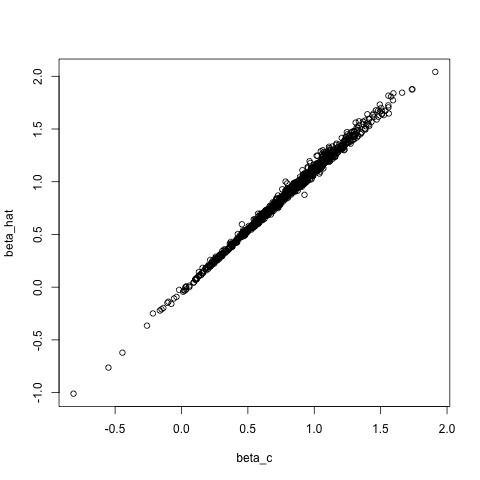

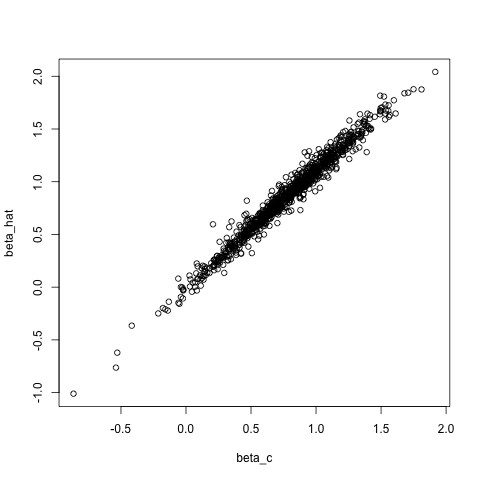

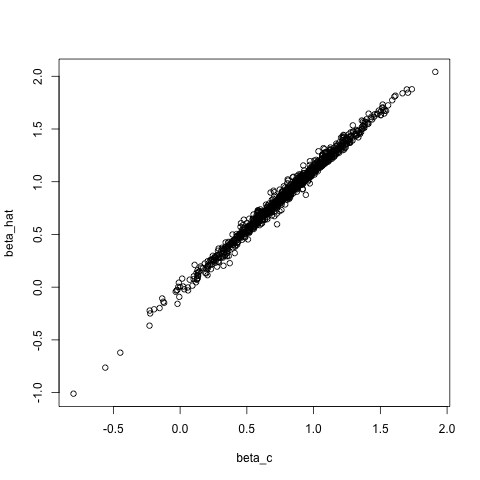


(a) full data, MAF=2% (b) subset data, MAF=2% (c) random, MAF=2%


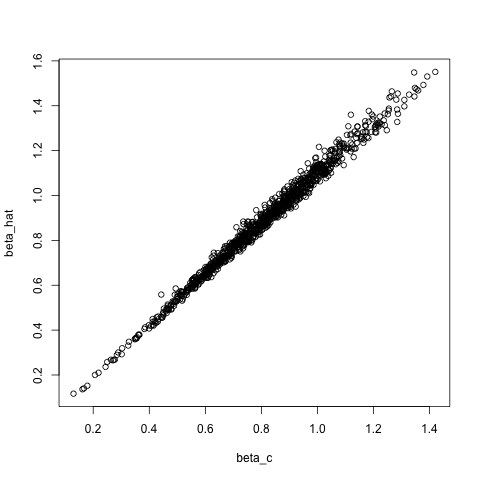

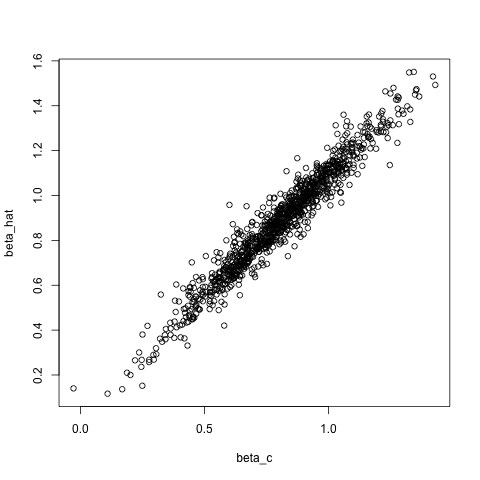

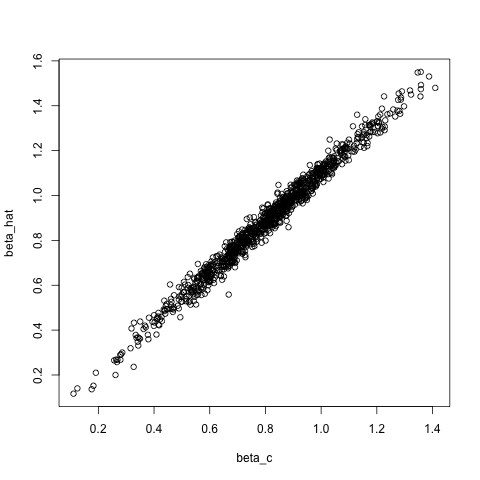


(d) full data, MAF=5% (e) subset data, MAF=5% (f) random, MAF=5%


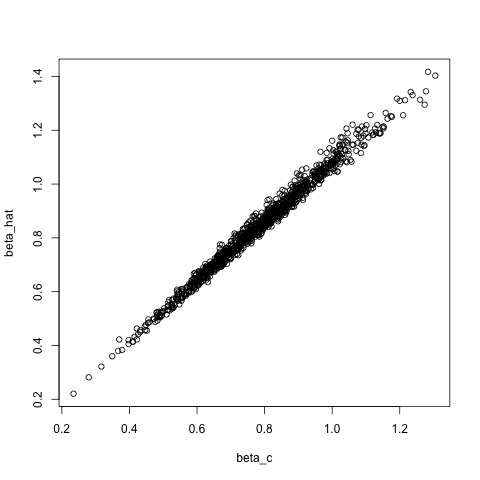

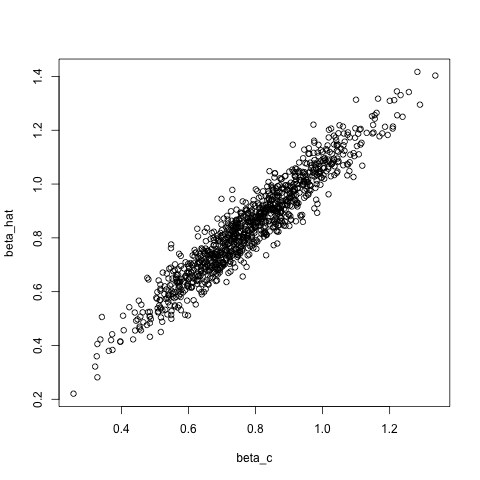

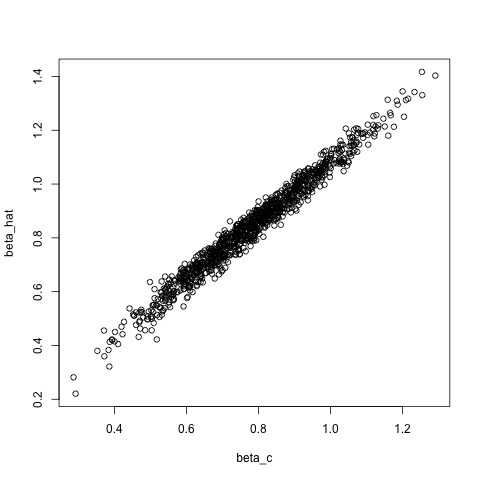


(g) full data, MAF=10% (h) subset data, MAF=10% (i) random, MAF=10%


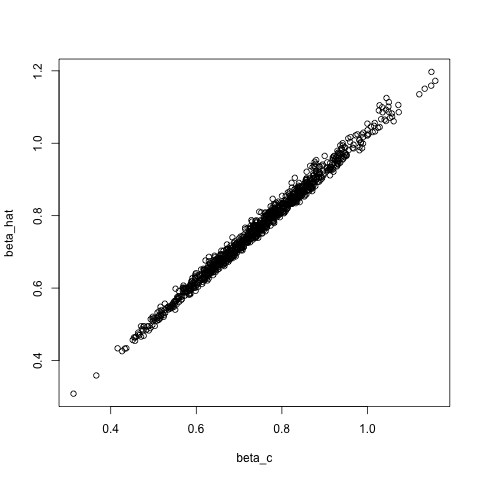

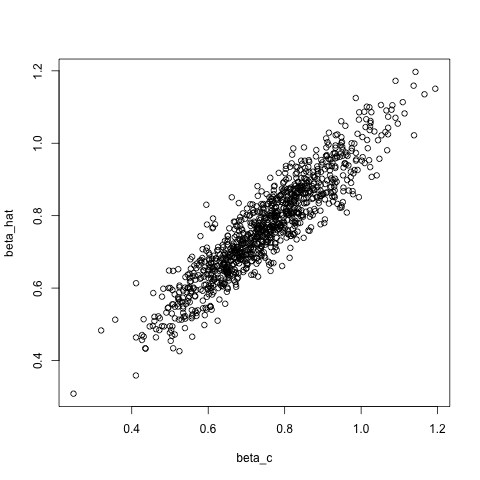

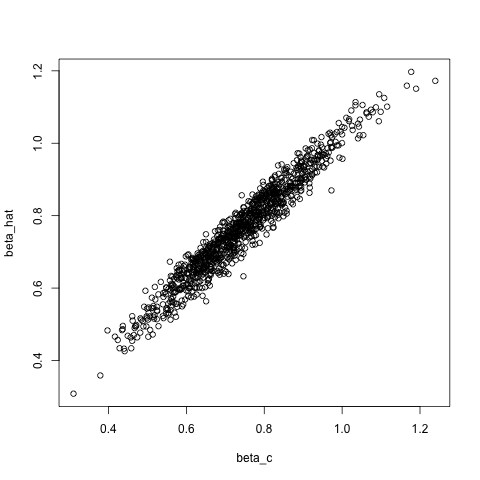


(j) full data, MAF=25% (k) subset data, MAF=25% (l) random, MAF=25%

Supplementary Figure 7: Beta estimates for two binary traits. “beta c” means beta estimates calculated by our proposed method. “beta hat” means beta estimates calculated by individual level data (gold standard)


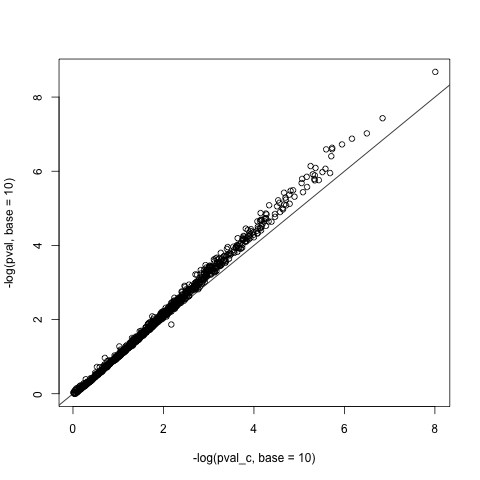

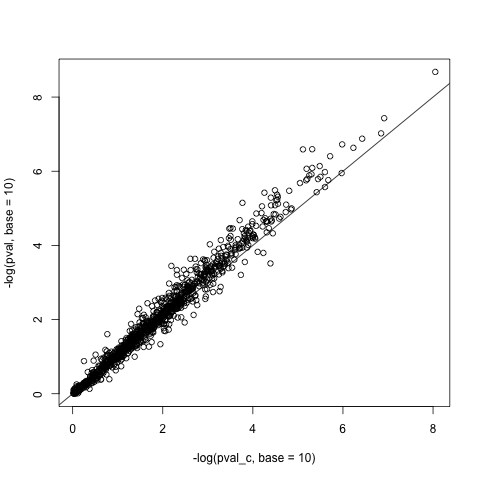

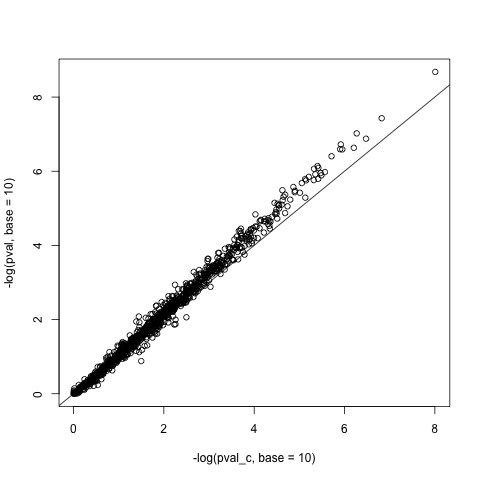


(a) full data, MAF=2% (b) subset data, MAF=2% (c) random, MAF=2%


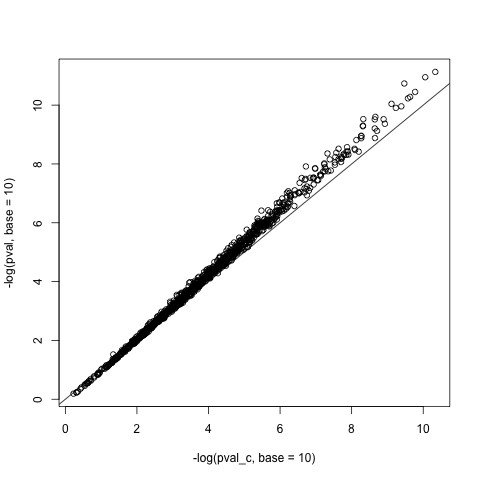

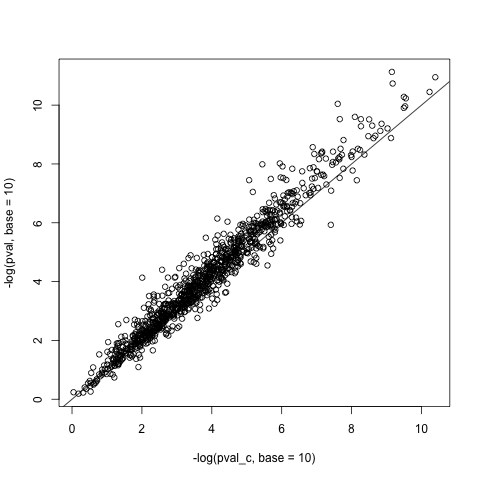

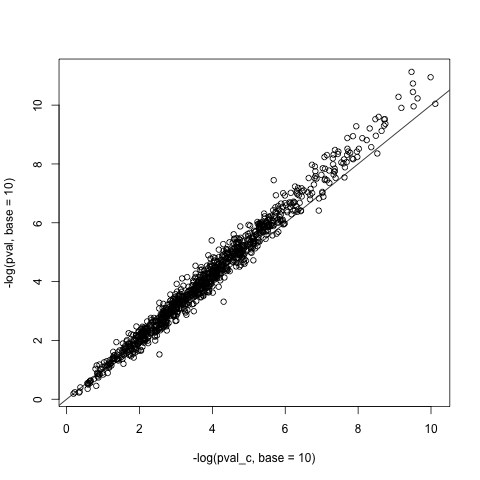


(d) full data, MAF=5% (e) subset data, MAF=5% (f) random, MAF=5%


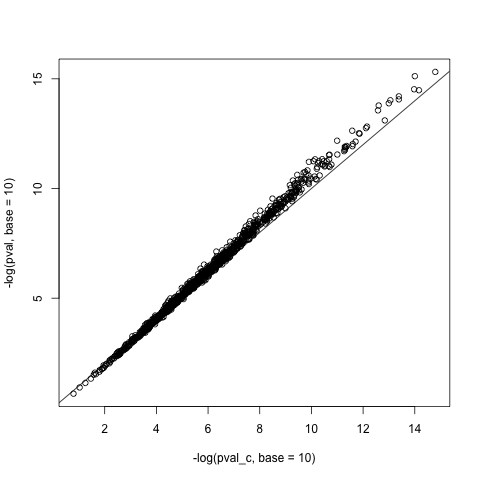

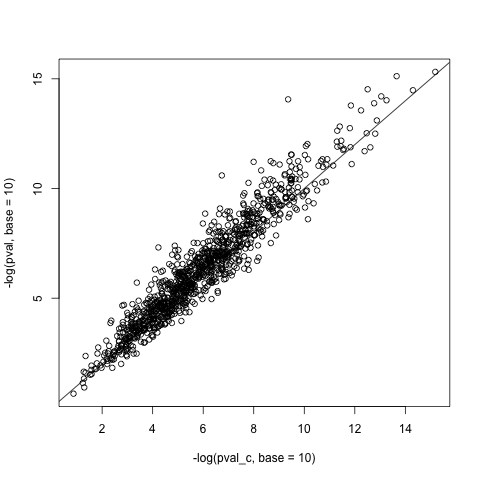

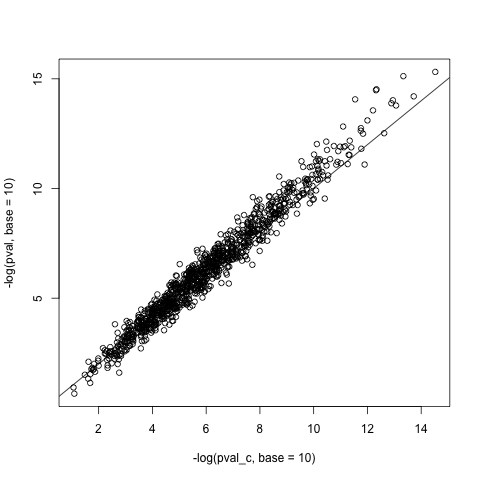


(g) full data, MAF=10% (h) subset data, MAF=10% (i) random, MAF=10%


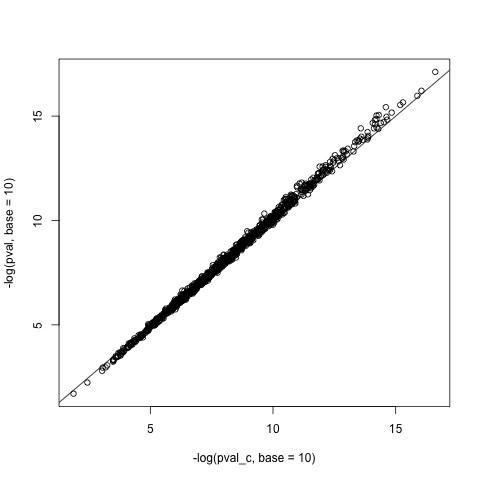

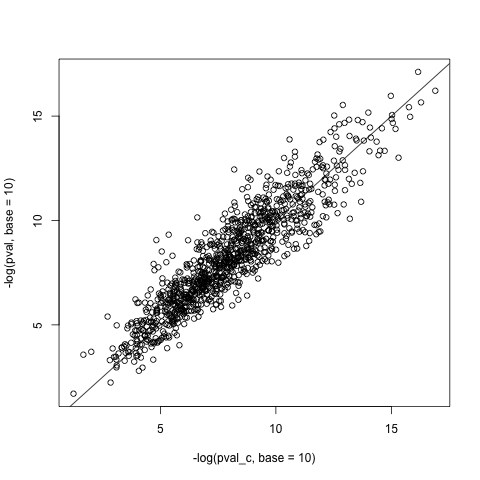

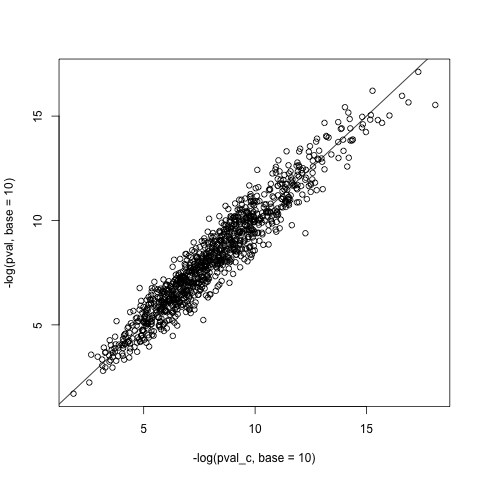


(j) full data, MAF=25% (k) subset data, MAF=25% (l) random, MAF=25%

Supplementary Figure 8: *p*-values for two binary traits. “pval c” means *p*-values calculated by our proposed method. “beta hat” means *p*-values calculated by individual level data (gold standard)


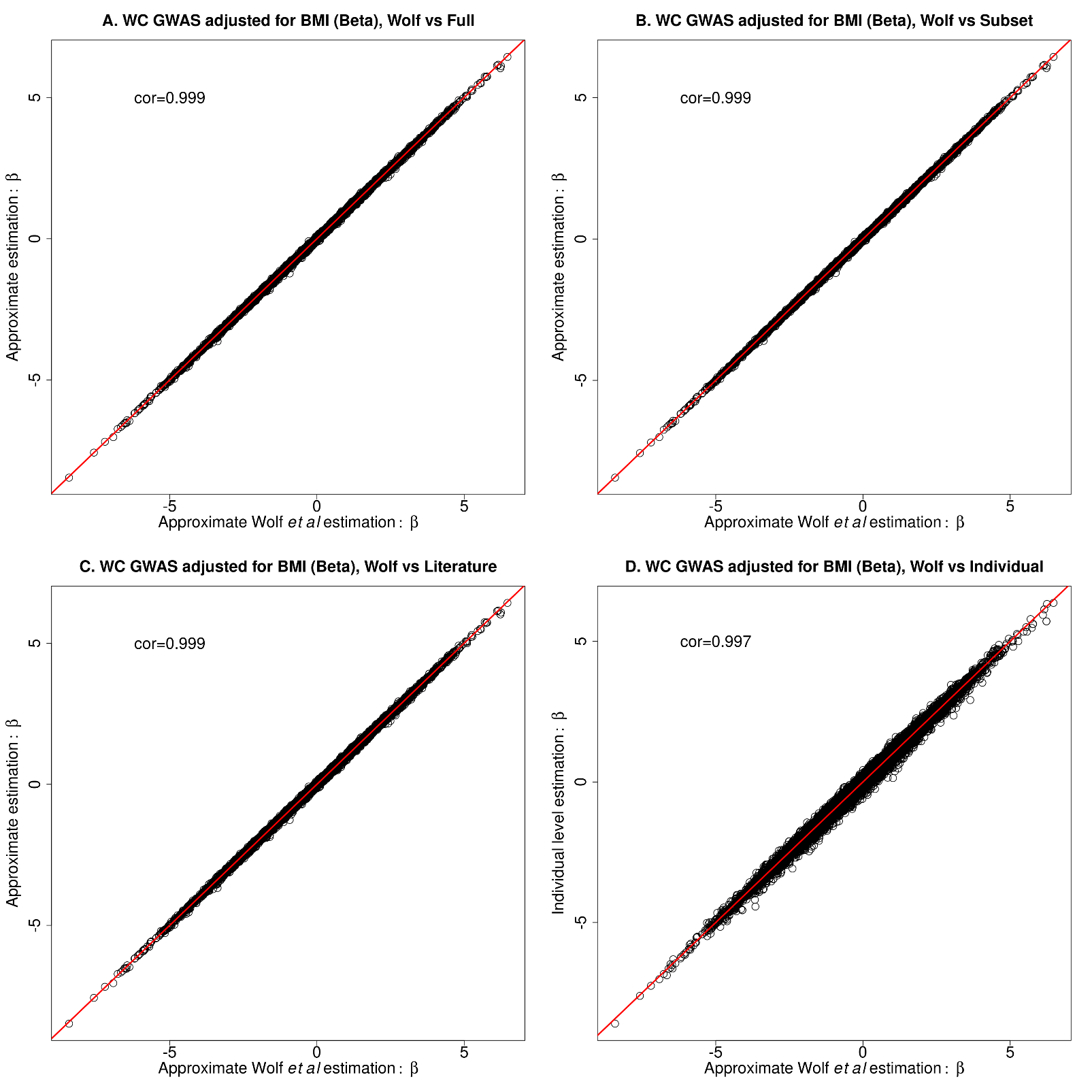


Supplementary Figure 9: Effect size estimates for our proposed method and for Wolf et al method using the Framingham Heart Study. “Cor” means correlation coefficient.


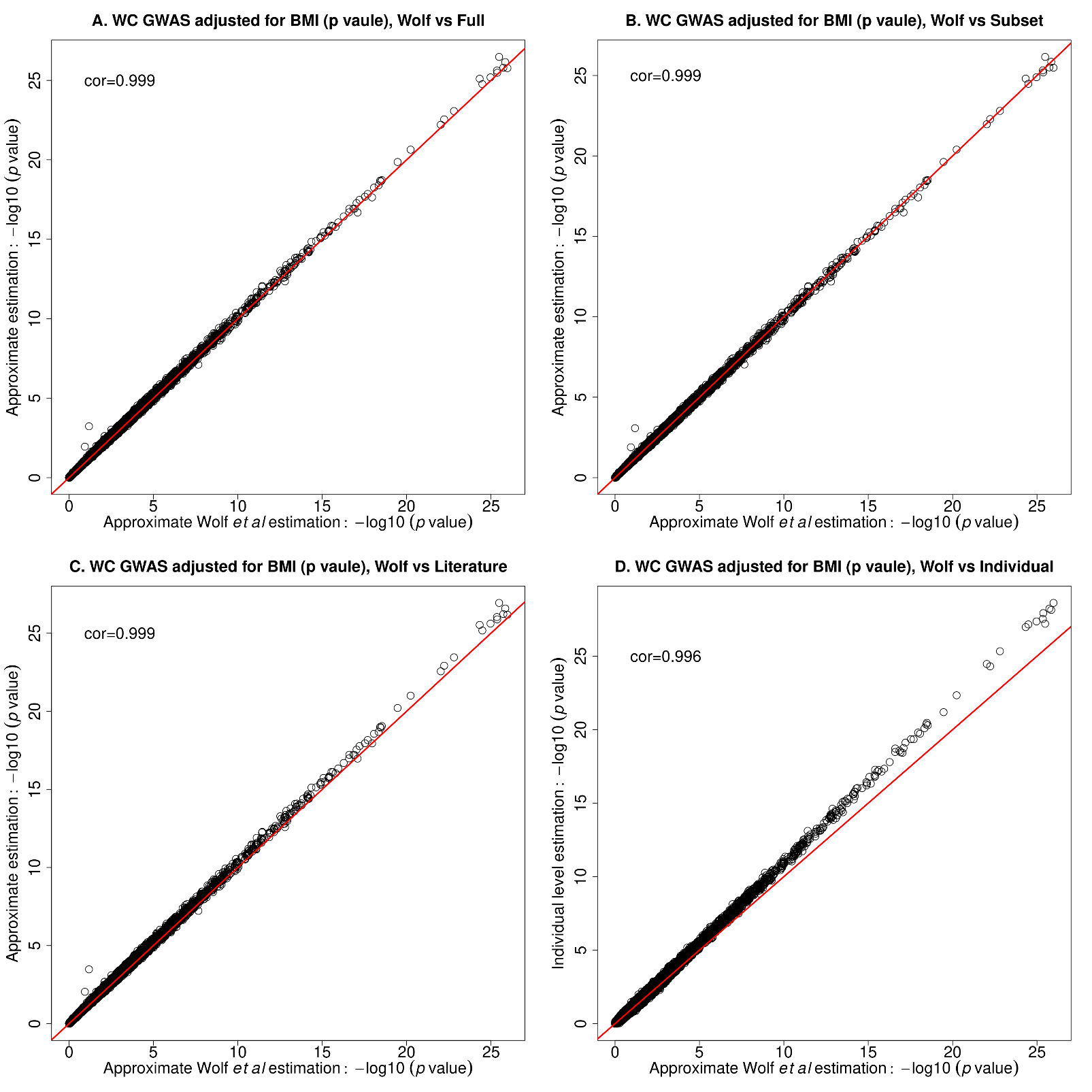


Supplementary Figure 10: *P* value for our method and for the Wolf et al method using the Framingham Heart Study. “Cor” means correlation coefficient.

| **Outcome is continuous, covariate is binary (BMI GWAS adjusted for ever smoked)** | |
| --- | --- |
| 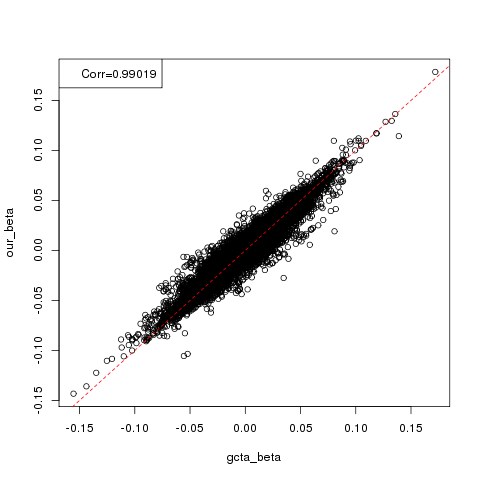 | 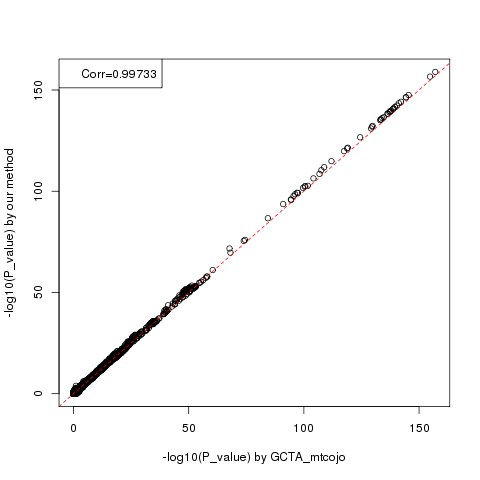 |
| **Outcome is binary, covariate is continuous (AF GWAS adjusted for BMI)** | |
|  |  |
| **Two binary traits (AF GWAS adjusted for CHD)** | |
|  |  |

Supplementary Figure 11: Comparing our method with GCTA multi-trait conditional and joint analysis (GCTA mtCOJO). “Corr” means correlation coefficient.

Supplementary Figure 12: Comparison of effect sizes and p-values for the application in existing GWAS meta-analysis for fasting insulin adjusted for body mass index when using the mean minor allele frequencies across consortia: Individual level data is the gold standard for estimation, “gcta” means we used multi-trait-based conditional and joint analysis (mtCOJO) provided by GCTA 1.9 (GCTA mtCOJO), with the Framingham Heart Study (FHS) unrelated individuals as LD reference panel. “ours” means our method and the phenotype data from FHS for estimation the relationship between traits.

Supplementary Figure 13: Comparison of effect sizes and p-values for the application in existing GWAS meta-analysis for fasting insulin adjusted for body mass index when filtering out variants with significant difference in minor allele frequencies between the consortia: Individual level data is the gold standard for estimation, “gcta” means multi-trait-based conditional and joint analysis (mtCOJO) provided by GCTA 1.9 (GCTA mtCOJO) with the Framingham Heart Study (FHS) unrelated individuals as LD reference panel. “ours” means our method and the phenotype data from FHS for estimation the relationship between traits.

Supplemental Figure 14. Comparisons of p values and number of genome-wide significant SNPs for our method with and without Q_homo_ correction for the analysis of WC adjusted for BMI in the Framingham Heart Study.

Approximation Q_homo_ estimation (or “_Qhomo”) indicates results corrected using the Wang et al approach. The individual level estimation represents covariate adjusted results using individual level data, “full” means the relationship between the outcome and the covariate is estimated using the full sample, “subset” means the relationship is estimated using a random sample of 1,000 individuals, “litt.” or literature means the relationship is taken from published reports. ^1^

Genome-wide significant level =0.05/total number of variants.

Reference

1. Bozeman, S. R. *et al.* Predicting waist circumference from body mass index. *BMC Med. Res. Methodol.* **12**, (2012).
